# Human tau-overexpressing mice recapitulate brainstem involvement and neuropsychiatric features of early Alzheimer’s disease

**DOI:** 10.1186/s40478-023-01546-5

**Published:** 2023-04-03

**Authors:** Kanza M. Khan, Nagalakshmi Balasubramanian, Gabriel Gaudencio, Ruixiang Wang, Govindhasamy Pushpavathi Selvakumar, Louis Kolling, Samantha Pierson, Satya M. Tadinada, Ted Abel, Marco Hefti, Catherine A. Marcinkiewcz

**Affiliations:** 1 Department of Neuroscience and Pharmacology, University of Iowa, 2-430 Bowen Science Building, Iowa City, IA 52242 USA; 2 Department of Pathology, University of Iowa, Iowa City, IA 52242 USA; 3 Psychological Sciences Department, Daemen University, Amherst, NY 14226 USA

**Keywords:** Alzheimer’s disease, Tau, Depression, Htau, Serotonin, Norepinephrine

## Abstract

Alzheimer’s disease (AD) poses an ever-increasing public health concern as the population ages, affecting more than 6 million Americans. AD patients present with mood and sleep changes in the prodromal stages that may be partly driven by loss of monoaminergic neurons in the brainstem, but a causal relationship has not been firmly established. This is due in part to a dearth of animal models that recapitulate early AD neuropathology and symptoms. The goal of the present study was to evaluate depressive and anxiety-like behaviors in a mouse model of AD that overexpresses human wild-type tau (htau) prior to the onset of cognitive impairments and assess these behavior changes in relationship to tau pathology, neuroinflammation, and monoaminergic dysregulation in the dorsal raphe nucleus (DRN) and locus coeruleus (LC). We observed depressive-like behaviors at 4 months in both sexes and hyperlocomotion in male htau mice. Deficits in social interaction persisted at 6 months and were accompanied by an increase in anxiety-like behavior in males. The behavioral changes at 4 months coincided with a lower density of serotonergic (5-HT) neurons, downregulation of 5-HT markers, reduced excitability of 5-HT neurons, and hyperphosphorylated tau in the DRN. Inflammatory markers were also upregulated in the DRN along with protein kinases and transglutaminase 2, which may promote tau phosphorylation and aggregation. Loss of 5-HT innervation to the entorhinal cortex and dentate gyrus of the hippocampus was also observed and may have contributed to depressive-like behaviors. There was also reduced expression of noradrenergic markers in the LC along with elevated phospho-tau expression, but this did not translate to a functional change in neuronal excitability. In total, these results suggest that tau pathology in brainstem monoaminergic nuclei and the resulting loss of serotonergic and/or noradrenergic drive may underpin depressive- and anxiety-like behaviors in the early stages of AD.

**Supplementary Information:**

The online version contains supplementary material available at 10.1186/s40478-023-01546-5.

## Introduction

Alzheimer’s disease (AD) is a devastating age-related neurodegenerative disease that afflicts a large proportion of individuals aged 65 and older[1]. Recent evidence suggests that neurofibrillary tangles (NFT) develop in brainstem nuclei including the dorsal raphe nucleus (DRN) and locus coeruleus (LC) before the hippocampus and cortex, which may lead to loss of monoaminergic neurons and neuropsychiatric symptoms (NPS) in the prodromal stages of AD [2–13]. The DRN contains a large population of serotonin (5-HT) neurons that project to the forebrain and regulate mood, sleep, and reward-seeking behaviors, all of which are perturbed in AD [14–19]. The LC is also associated with depressive and anxiety-like behaviors in both humans and rodent models [20, 21], with studies suggesting that even a minimal loss of noradrenergic (NA) neurons can lead to depressive behavior [22].

Perturbations in brainstem monoaminergic nuclei may drive prodromal neuropsychiatric symptoms in AD, but direct evidence linking brainstem neuropathology and monoaminergic depletion to specific behavioral changes in early AD is currently lacking. AD is usually diagnosed at later stages when significant neurodegeneration has already taken place, so there is a critical need to develop and characterize model systems that recapitulate the early stages of AD to help identify new biomarkers and therapeutic targets. The htau mouse model is a genetic cross between a mouse microtubule-associated protein tau (*MAPT*) tau knockout line [23] and the 8c line [24] that contains a wild-type human *MAPT* transgene under the tau promoter that results in expression of all six isoforms of human tau. These mice develop tau pathology in a more naturalistic fashion such that hippocampal dysfunction and memory deficits occur relatively late in life around 12 months of age [25]. These mice are cognitively intact at 4 months of age, but there is evidence of hyperphosphorylated tau in the DRN [26] which may be associated with altered serotonergic function and behavioral dysregulation reminiscent of prodromal AD. The goal of the present study was to develop a behavioral and neurochemical profile of htau mice at 4–6 months of age and determine whether they might be a useful model of prodromal AD. We first assessed htau mice for depressive and anxiety-like behaviors using a battery of tests including the elevated plus maze (EPM) and elevated zero maze (EZM), open field, social interaction test, sucrose preference test, and forced swim test. Monoaminergic neurons and glia in the DRN and LC were examined by immunohistochemistry, and ex vivo electrophysiology was used to assess functional changes in 5-HT and NA neurons. Expression of genes involved in monoamine biosynthesis and signaling, neuroinflammation, and proteostasis were also examined in brainstem tissues. Finally, serotonergic inputs to the entorhinal cortex (EC) and hippocampus may also be impacted by brainstem tau pathology and have been implicated in affective and cognitive changes in AD [27, 28]. We examined 5-HT immunoreactivity in the EC and serotonin transporter (SERT) immunoreactivity in the hippocampus, as well as mRNA expression of 5-HT receptors, tau-related genes, and inflammatory markers in these regions. Overall, our results suggest that tau accumulation in the brainstem coincides with monoaminergic dysfunction and NPS in the early stages of AD. Additionally, htau mice may be a useful model for testing therapeutic interventions aimed at ameliorating tau pathology in the brainstem and arresting neurodegeneration in early AD.

## Materials and methods

### Animals

Male and female C57BL/6J mice (Jackson Labs #000664) and htau +/- mice (Jackson Labs #005491) containing a transgene that encodes the human *MAPT* gene were used in this experiment. *MAPT* -/- (global tau knockout; Mapt < tm1(EGFP)Klt) mice were used as negative controls in RT-PCR and Western blot validation of tau splice isoforms. Mice were housed in a temperature-and humidity-controlled, AALAC-approved vivarium at the University of Iowa with ad libitum access to food and water.

### Behavior

**Fig. 1:**
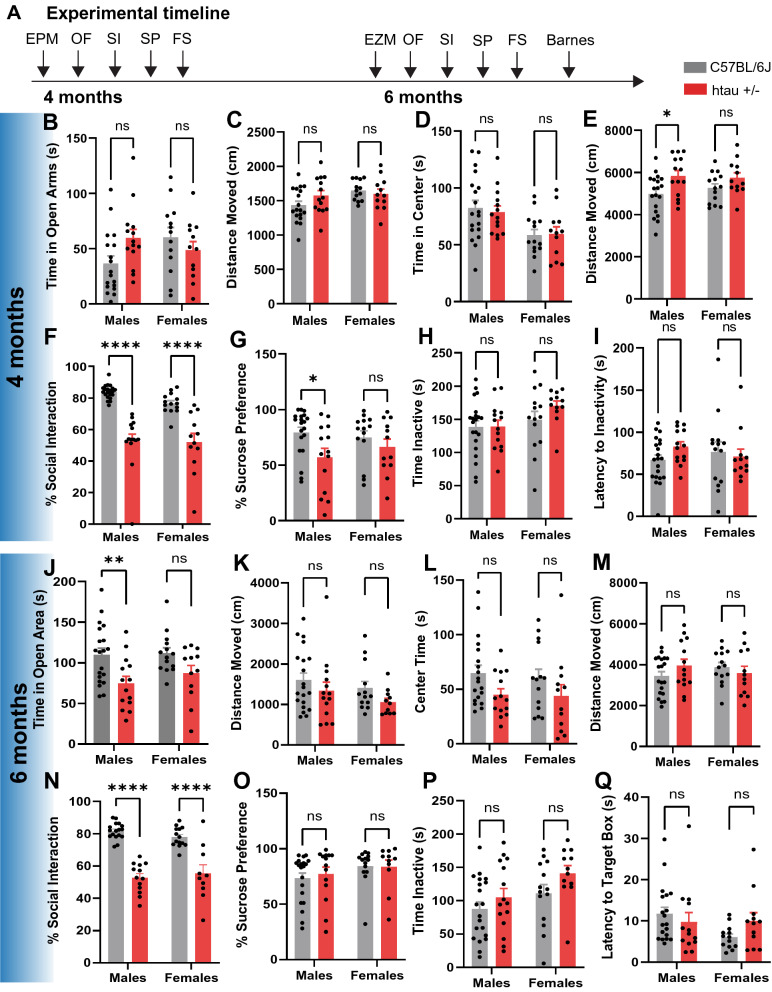
Htau mice exhibit depressive-like behaviors at 4 and 6 months of age. **A** Experimental timeline of behavioral studies in htau and C57BL/6J mice. Behavior of 4-month-old htau and C57BL/6J mice in the **B, C** EPM, **D**, **E** open field, **F** social interaction test, **G** sucrose preference test and **H**, **I** forced swim test. Behavior of 6-month-old htau and C57BL/6J mice in the **J**, **K** EZM, **L**, **M** open field, **N** social interaction test, **O** sucrose preference test, **P** forced swim test and **Q** Barnes maze. These results indicate depressive-like behaviors in htau mice relative to C57BL/6J mice. *p < 0.05, **p < 0.01, ****p < 0.0001

Anxiety- and depressive-like behaviors in C57BL/6J and htau +/- mice were evaluated at 4 and 6 months of age. The order in which behavioral tests were performed is depicted in Fig. 1A. All behavior tests were performed during the light phase of the light/dark cycle (9 am–3 pm) and were recorded with an overhead or side-view camera integrated with Media Recorder or Ethovision video tracking software (Noldus Information Tech, Inc.). Videos were scored by an experimenter blinded to animal genotypes. Unless otherwise noted, all behavior tests were scored using Ethovision.

#### Elevated plus maze

At 4 months of age, animals were tested in the EPM to evaluate anxiety-like behaviors [29]. Briefly, the maze was 60 cm above the floor and consisted of two open arms, two closed arms (5 × 35 cm), and a neutral starting zone (5 × 5 cm). Overhead LEDs were used to maintain lux in the open arms at 20 lux and < 5 lux in the closed arms. The closed arms had tall dark walls that allowed animals to hide. At the beginning of the test, animals were placed in the neutral zone and allowed to freely explore the maze for 5 min. Distance traveled, and time spent in the open arms vs closed arms were calculated using Ethovision XT14.

#### Elevated zero maze

At 6 months of age, animals were tested in the EZM to evaluate anxiety-like behaviors in a novel arena [30–32]. Briefly, the circular maze (outer diameter: 52 cm) was elevated 60 cm above the ground and consisted of two alternating open and closed corridors (width: 5 cm). Overhead LEDs maintained the lux in the open corridors at 20 lux and < 5 lux in the closed corridors. At the beginning of the test, animals were placed in the open corridor, facing the closed corridor, and allowed to freely explore the arena for 5 min. Behavior was recorded by an overhead camera. Distance traveled, and time spent in the open area vs closed corridors were calculated using Ethovision XT14. The open area preference and probability of entering the open areas were calculated to determine the anxiety-like behavior.

#### Open field test

Mice were placed in the corner of a 50 × 50 × 25 cm opaque plexiglass arena (20 lux) and allowed to freely explore the arena for 30 min. The open field test was performed at 4 and 6 months of age to evaluate locomotor and exploratory behavior. The total distance traveled (cm), time spent in the center of the arena, and time spent in the corners of the arena were measured through Ethovision XT14. The center of the open field was defined as the central 15% of the arena.

#### Social interaction test

The social interaction test was performed in 4- and 6-month-old mice, and was performed as previously described [33, 34]. Briefly, the animal was placed in the central chamber of a transparent 3-chamber arena (20 lux) and allowed to explore the environment for 10 min. Following this, a novel C57BL/6J mouse (stranger mouse) of the same sex and approximate age as the experimental mouse was placed in one of the side chambers under a metal cage. An empty metal cage was also placed in the alternate side chamber. The experimental mouse was allowed to explore the environment and interact with the stranger mouse for 10 min. The location of the stranger mouse was alternated between the right and left chamber to control for any side preferences. The total time spent interacting with the stranger mouse and the empty cage was scored.

#### Sucrose preference test

The sucrose preference test was performed over four days and used to evaluate the degree of anhedonia of htau mice at 4 and 6 months of age [35, 36]. Animals were transferred to PhenoTyper observation cages (Noldus) that were fitted with two sipper bottles and Lickometers (to measure the number of licks the animal makes to a bottle). Animals had ad libitum access to tap water or a 5% sucrose solution (ThermoFisher) for 1 h. The placement of sucrose and water bottles was alternated each day to control for any side preference. The number of approaches to the water bottle and the sucrose bottle was measured through Ethovision XT14.

#### Barnes maze

Spatial learning and cognitive deficits were evaluated in 6-month-old C57BL/6 J and htau mice as described previously [32, 37, 38]. The Barnes maze was a 5-day protocol [39], with environment habituation on day 1, training sessions on days 2 and 3, rest on day 4, and a probe trial on day 5. The maze was a gray circular arena (diameter: 91 cm), consisting of 20 equally divided holes, and was elevated 93 cm above the ground. The room was well lit and visual cues were present on the walls.

On Day 1, animals were guided to the predetermined ‘goal box’, which had been fitted with an escape chamber. In contrast, the remaining 19 (non-target holes) were not fitted with any chambers, and animals could see the ground below.

Over days 2 and 3, animals underwent a total of five training trials. A buzzer sound (~ 100 dB) was played while animals explored the environment. Once the animal found the goal box and entered the escape chamber, the buzzer was turned off and animals were allowed to rest for 1-min before being returned to a holding cage. If an animal did not find the goal box/escape chamber within a 2-min trial, they were guided to the escape chamber as they had been on Day 1. Inter-trial interval was 30 min. Animals had 3 training trials on day 2, and 2 trials on day 3.

On the probe day, the escape chamber was removed from the goal box. Animals were placed on the maze platform, and the buzzer sound was presented. Animals were allowed to explore the environment for 2 min. Behavior was recorded by an overhead camera.

The number of visits to the goal box & non-target holes was measured along with the latency to approach the goal box and time spent in the target quadrant.

#### Forced swim test

Depressive-like behavior was evaluated at 4 and 6 months in the forced swim test. Mice were gently placed in a tall cylinder filled with 24–25 °C tap water (32 cm height × 20 cm diameter, water height: 25 cm) for 6 min. After the test, animals were placed in a clean cage under a heating lamp for 5 min to warm them and allow them to dry off. Animal behavior was recorded from a side-view camera and analyzed with Ethovision XT14. The 6-min videos were divided into two bouts: a pretest (first 1–2 min) and a test (last 3–6 min) phase. The latency to the first immobile bout, frequency of immobile bouts, and duration of each immobile bout was evaluated.

### Immunofluorescence

A cohort of C57BL/6 J and htau +/- mice were deeply anesthetized with tribromoethanol and transcardially perfused with PBS followed by 4% paraformaldehyde (PFA) to collect brains for immunofluorescence experiments. Brains were cryosectioned at 25 µm using a Leica cryostat (CM3050S, Leica, Germany). Slices were stored at 4 °C in a cryoprotectant solution. The immunofluorescence was performed as described previously [40, 41]. Briefly, for each region of interest (ROI), 3–4 slices were used across the rostral-caudal axis. Slices were washed in PBS and incubated in 0.5% Triton X-100/PBS for 30 min, blocked in 10% normal donkey serum in 0.1% Triton X-100/PBS, and then incubated with the respective primary and secondary antibodies (Table 1). For AT8 IF experiments, mouse-on-mouse blocking reagent (3% final volume; Vector Laboratories) was added to the blocking solution to reduce non-specific binding to endogenous mouse IgG. Slices were subsequently washed in PBS, mounted on glass slides, and coverslipped with Vectashield mounting media (Vector Laboratories, Inc.).

**Table 1:** Antibodies used in immunohistochemistry

Stain	Antibody	Concentration
5HT-AT8	Goat anti-5HT (Immunostar; catalog # 20079)	1:2000
	Mouse anti-AT8 (Thermofisher, catalog # MN1020)	1:200
	405 Donkey anti-Goat (Jackson ImmunoResearch; catalog # 715-475-003)	1:500
	Cy3 Donkey anti-Mouse (Jackson ImmunoResearch; catalog # 711-165-151)	1:500
TH-AT8	Rabbit anti-TH (Novus; catalog # NB300-109)	1:1000
	Mouse anti-AT8 (Thermofisher, catalog # MN1020)	1:200
	405 Donkey anti-Rabbit (Abcam; catalog # ab175651)	1:500
	Cy3 Donkey anti-Mouse (Jackson ImmunoResearch; catalog # 715-165-151)	1:500
5HT-iba1-GFAP	Goat anti-5HT (Immunostar; catalog # 20079)	1:2000
	Rabbit anti-iba1 (Abcam; catalog # ab178846)	1:200
	Chicken anti-GFAP (Abcam; catalog # ab4674)	1:6000
	405 Donkey anti-Goat (Jackson ImmunoResearch; catalog # 715-475-003)	1:500
	Cy3 Donkey anti-Rabbit (Jackson ImmunoResearch; catalog # 711-165-152)	1:500
	647 Donkey anti-Chicken (Jackson ImmunoResearch; catalog # 703-605-155)	1:500
TH-iba1-GFAP	Mouse anti-TH (Millipore Sigma; catalog # MAB318)	1:200
	Rabbit anti-iba1 (Abcam; catalog # ab178846)	1:200
	Chicken anti-GFAP (Abcam; catalog # ab4674)	1:6000
	405 Donkey anti-Mouse (Invitrogen; catalog # A48257)	1:500
	Cy3 Donkey anti-Rabbit (Jackson ImmunoResearch; catalog # 711-165-152)	1:500
	647 Donkey anti-Chicken (Jackson ImmunoResearch; catalog # 703-605-155)	1:500
SERT	Rabbit anti-SERT (Immunostar; catalog #24330)	1:2500
	Alexa Fluor 555 donkey anti-rabbit (Invitrogen; catalog # A31572)	1:500

Confocal z-stacks (1 µm) were captured on an Olympus FV3000 laser scanning confocal microscope (20 sections/z-stack) and converted to maximum projection images using Image J software. Images were analyzed by trained researchers blind to experimental conditions to obtain cell counts per unit area, % immunoreactive area, and optical density using ImageJ. The optical density was estimated by first converting images to an 8-bit grayscale image and performing a background correction. Optical density calibration was performed with a 21-step tablet (available from ImageJ) using the Rodbard function. Following optical density calibration, mean grayscale values were recorded from the ROIs with an effort to avoid artifacts. Percent (%) immunoreactive area was performed on the ROIs of thresholded images. For each image, the ROI was drawn according to the shape of the region based on the mouse brain reference atlas of Paxinos & Franklin [42] and the monoaminergic signal. ROIs of the nuclei were drawn based on the reference atlas and IF immunoreactivity for each image, and then applied to all channels (AT8, Iba-1, or GFAP).

### Ex vivo electrophysiology

#### Brain slice preparation

Deeply anesthetized mice were transcardially perfused with ice-cold, oxygenated modified artificial cerebrospinal fluid (aCSF) containing the following (in mm): 110 choline-Cl, 2.5 KCl, 7 MgSO_4_, 0.5 CaCl_2_, 1.25 NaH_2_PO_4_, 26.2 NaHCO_3_, 25 glucose, 11.6 Na-ascorbate, 2 thiourea, and 3.1 Na-pyruvate (pH: 7.3–7.4; osmolality: 300–310 mOsmol/kg). Then their brains were quickly dissected and coronal slices containing the DRN or LC (300 μm) were obtained using a vibratome (VT1200S; Leica Biosystems, Wetzlar, Germany). The brain slices recovered at 34 °C for 30 min in a chamber containing the choline-Cl-based aCSF described above and continuously bubbled with 95% O_2_/5% CO_2_. After the initial recovery, brain slices were transferred to and held in a different modified aCSF at room temperature, saturated with 95% O_2_/5% CO_2_, for at least 1 h before recordings started. The holding aCSF contained the following (in mm): 92 NaCl, 2.5 KCl, 2 MgSO_4_, 2 CaCl_2_, 1.25 NaH_2_PO_4_, 30 NaHCO_3_, 20 HEPES, 25 glucose, 5 Na-ascorbate, 2 thiourea, and 3 Na-pyruvate (pH: 7.3–7.4; 300–310 mOsmol/kg).

#### Ex vivo electrophysiological recordings

During recordings, slices were continuously perfused (2 ml/min) with standard aCSF containing (in mm): 124 NaCl, 4 KCl, 1.2 MgSO_4_, 2 CaCl_2_, 1 NaH_2_PO_4_, 26 NaHCO_3_, and 11 glucose (300–310 mOsmol/kg), saturated with 95% O_2_/5% CO_2_ and maintained at 30 ± 1 °C. Patch electrodes (3–5 MΩ) were filled with a solution containing (in mm): 135 K-gluconate, 5 NaCl, 2 MgCl_2_, 10 HEPES, 0.6 EGTA, 4 Na_2_-ATP, and 0.4 Na_2_-GTP (pH: 7.3; 288–292 mOsmol/kg). In order to identify 5-HT or NA neurons post hoc by immunofluorescence, biocytin (2 mg/ml; Tocris Bioscience, Bristol, UK) was added into the internal solution. Only tryptophan hydroxylase 2 (TPH2)-positive neurons from the DRN and tyrosine hydroxylase (TH)-positive neurons from the LC were included in data analysis. Neurons were visualized via an upright microscope (BX51W1; Olympus, Tokyo, Japan) accompanied by a differential interference contrast imaging system. Membrane currents were amplified with a Multiclamp 700 B amplifier (Molecular Devices, San Jose, CA, USA), filtered at 3 kHz, and sampled at 20 kHz with a Digidata 1550B digitizer (Molecular Devices). Data were acquired via the pClamp 11 software (Molecular Devices). Access resistance was monitored online and changes greater than 20% would lead to discontinuation of the recordings.

To examine neuronal intrinsic excitability, recordings were conducted in current clamp mode. The DRN 5-HT neurons we patched were not spontaneously firing, and we performed recordings at resting membrane potential (RMP) and at − 70 mV holding potential to offset the variation of RMP. Input resistance was assessed by the change in membrane potential upon − 100 pA hyperpolarizing current injection. Rheobase reflected the minimal current needed to evoke action potentials (AP). Numbers of evoked APs were recorded after injecting depolarizing currents for 250 ms at 10 pA incremental steps (0–200 pA).

All the LC NA neurons were spontaneously firing, which we continuously recorded for 10 min and analyzed their firing profiles based on the last 5 min: firing frequency, mean amplitude of action potentials, variation of firing timing (assessed by coefficient of variation: CV = mean/standard deviation of inter-firing intervals). Because the NA neurons were spontaneously firing, it was impossible to assess their intrinsic excitability at RMP. Therefore, we slightly hyperpolarized the neurons by holding them at − 55 mV and performed the same recordings as in DRN 5-HT neurons.

AP characteristics were measured using pClamp, version 10 (Clampfit 10.7; Molecular Devices; RRID: SCR_011323). Many AP parameters require a clearly defined baseline in order to be assessed accurately, and the parameters are dependent on current injection magnitude. Due to differences in excitability between neurons, the current injection steps of 120 pA (held at − 70 mV) for the DR and 140 pA (held at − 55 mV) for the LC had the largest sample size for the comparison of AP characteristics that are dependent upon verifiable baseline. The metrics of ‘maximum decay slope’ and ‘time to achieve maximum decay slope’ are not dependent upon the correct identification of the recording baseline, so we examined these metrics simultaneously across all current injection steps.

### Reverse transcriptase-quantitative PCR

DRN, LC, EC, dorsal hippocampus (DHP) and ventral hippocampus (VHP) tissues were micro-punched from C57BL/6J and htau +/- brains, and total RNA was isolated as described previously [41]. Briefly, the RNA was extracted from the tissue using the TRIzol reagent method. The DNA contaminants from the extracted RNA were eliminated using a DNA-free™ DNA Removal Kit (Life Technologies, USA). The concentration and purity of RNA were checked using a NanoDrop 1000 spectrophotometer and the RNA was reverse transcribed to cDNA. RT-qPCR for the target genes was performed using SYBR green qPCR master mix (Bio-Rad Laboratories, USA) and specified primers (Table 3) on a CFX96™ Real-time-PCR System (Bio-Rad Laboratories, USA). The thermal profile used for RT-qPCR was 95 °C for 10 min, 40 cycles of 95 °C for 30 s, 60 °C for 30 s, followed by a melt curve analysis profile (60 °C to 95 °C in 0.5 °C increments at a rate of 5 s/step). Fold changes in the mRNA levels were determined for each gene after normalizing with β-actin Ct values using the fold change 2^−ΔΔCT^ method [43]. Results are represented as fold changes in the mRNA levels (± SEM).

### Western blot

A separate group of C57BL/6J and htau +/- mice were decapitated under isoflurane anesthesia to collect brains for Western blot experiments as reported [41]. Briefly, DRN and LC brain regions were dissected for Western blots of ptau (AH36; pSer^202^/pThr^205^), total tau (HT7), TPH2, TH, indoleamine 2,3 dioxygenase 1 (IDO1), transglutaminase 2 (TGM2), β-actin, and GAPDH. Total proteins were isolated using RIPA buffer supplemented with protease and phosphatase inhibitors. Proteins were quantified using the BCA method and an equal amount of protein was resolved in 10% SDS-PAGE gel and transferred to a PVDF membrane (0.2 µm; Millipore) for immunoblotting. The blots were blocked with Starting Block T20 (TBS) Blocking Buffer (ThermoFisher Scientific) for 20 min at 37 °C and incubated overnight with primary antibodies specific to AH36, HT7, TPH2, TH, IDO1, TGM2, β-actin, and GAPDH at 4 °C (Table 2). Further, the blots were incubated with fluorescent or HRP-conjugated secondary antibodies (Table 2) as per the manufacturer's instructions. The blots were imaged and acquired using the LI-COR Odyssey Imager (LI-COR Inc.) at 700 and 800 nm. Protein bands were quantified using ImageJ software (National Institutes of Health) and the average relative density of the TPH2, TH, IDO1, and TGM2 was determined after normalization to β-actin or GAPDH. Results are represented as a mean relative density of the protein levels (± SEM).

**Table 2:** Antibodies used in Western blot

Antibodies	Use	Catalog details	Company	Dilution
AH36	Primary	SMC-601D	StressMarq Biosciences	1:2000
HT7	Primary	MN1000	Invitrogen	1:10000
TPH2	Primary	51124	Cell signaling technology	1:2000
TH	Primary	MAB318	Sigma-Aldrich	1:2000
β-actin	Primary	47778	Santa Cruz	1:10000
GAPDH	Primary	2118	Cell Signaling Technology	1:10000
IDO1	Primary	sc-137012	Santa Cruz	1:1000
TGM2	Primary	3557	Cell Signaling Technology	1:5000
Donkey-anti-Rabbit-IRDye 680RD	Secondary	P/N: 926-68073	Li-Cor	1:14000
Donkey-anti-Mouse-IRDye 800CW	Secondary	P/N: 926-32212	Li-Cor	1:14000
Donkey-anti-Rabbit-HRP	Secondary	A16023	ThermoFisher	1:5000
Donkey-anti-Mouse-HRP	Secondary	A16011	ThermoFisher	1:5000

### Statistical analysis

All data were analyzed with GraphPad Prism version 9 (GraphPad Software Inc, La Jolla, CA). Outliers were identified and removed using a ROUT test (Q = 1%). Experiments comparing two groups were analyzed with a Student’s *t-test*, with *p* < 0.05. Where variance between groups was unequal, a Welch’s *t-test* was performed (*p* < 0.05). Behavior experiments were analyzed using a two-way ANOVA and Bonferroni corrections for post hoc analyses. Data are reported and graphically represented as means ± standard error of the mean (SEM).

## Results

### Htau mice exhibit depressive-like behaviors by 4 months and anxiety-like behaviors by 6 months of age

In this study, we used htau +/- mice (Jackson Labs strain # 005491) that had been backcrossed to a mouse tau knockout line (Mapt < tm1(EGFP)Klt). These mice are on a C57BL/6J background, so C57BL/6J mice were used as controls throughout this study as in previous reports [25, 26, 44]. Htau mice express all six isoforms of human tau including the 3R and 4R isoforms and are thought to represent a more naturalistic model of AD with late-onset cognitive impairment [25] (Additional file 1: Fig. S1). We asked whether htau mice exhibit behavioral phenotypes reminiscent of prodromal AD by 4 months of age, which coincides with the appearance of hyperphosphorylated tau in the DRN but prior to the onset of cognitive impairments [25, 26]. Male and female htau and C57BL/6J mice were tested in the EPM, open field, social interaction test, sucrose preference test, and forced swim test at this time point (Fig. 1A). We found a significant interaction between sex and genotype on time spent in the open arms of the EPM at 4 months (F_1,53_ = 4.92, *p* < 0.05), but post-hoc comparisons between htau and C57BL/6J mice were non-significant in both males and females. No group differences in locomotor activity were observed in this assay (Fig. 1B, C). There was a main effect of sex in time spent in the center of the open field (F_1,55_ = 12.25, *p* < 0.001) but no sex x genotype interaction, suggesting that female mice generally exhibited more anxiety-like behavior in this test. Htau mice also exhibited hyperlocomotion in the open field (Main effect genotype: F_1,55_ = 8.32, *p* < 0.01) although Bonferroni post-tests were only significant in males (t_55_ = 2.77, *p* < 0.05) (Fig. 1D, E). Interestingly, both sexes exhibited significant depressive-like behaviors in the social interaction test (Main effect of genotype: F_1,57_ = 74.37, *p* < 0.0001; Bonferroni post-tests t_57_ = 7.32, *p* < 0.01 for males and t_57_ = 5.06, *p* < 0.0001 for females) (Fig. 1F), whereas sucrose preference was only reduced in male mice (Main effect genotype: F_1,56_ = 5.89, *p* < 0.05; Bonferroni post-test: t_56_ = 2.65, *p* < 0.05) (Fig. 1G). Overall, there was no effect of genotype on immobility time or latency to immobility in the forced swim test, but female mice spent more time immobile than males (Main effect of sex: F_1,57_ = 4.22, *p* < 0.05) (Fig. 1H, I).

We then examined anxiety and depressive-like behaviors in these mice at 6 months of age. Here we found a significant effect of genotype in time spent in the open area of the EZM (F_1,56_ = 12.21, *p* < 0.001), with male htau mice exhibiting a significant reduction in open area time (Bonferroni post-tests: t_56_ = 3.11, *p* < 0.01) (Fig. 1J). Locomotor activity did not significantly differ between groups in this test (Fig. 1K). There was also a significant effect of genotype in time spent in the center of the open field (F_1,55_ = 5.15, *p* < 0.05) that is suggestive of enhanced anxiety-like behavior, although post-hoc comparisons were non-significant in both sexes (Fig. 1L). Locomotor activity in the open field did not differ significantly between groups, which contrasted with the hyperlocomotive phenotype observed at 4 months (Fig. 1M). Depressive-like behaviors in the social interaction test persisted at 6 months in both sexes (Main effect of genotype: F_1,50_ = 94.73, *p* < 0.0001; Bonferroni post-tests: t_50_ = 8.23 for males and t_50_ = 5.70 for females) (Fig. 1N), although the decrease in the sucrose preference noted at 4 months had resolved by this time point (Fig. 1O). Similar to what we observed at 4 months, there was no effect of genotype in the forced swim test at 6 months, although female mice continued to spend more time inactive than males (Main effect of sex: F_1,57_ = 5.76, *p* < 0.05) (Fig. 1P). There was also no effect of sex or genotype on spatial memory in the Barnes Maze at 6 months (Fig. 1Q), which is consistent with previous reports that memory impairments in htau mice only become apparent at 12 months of age [25].

### Monoaminergic depletion and hyperphosphorylated tau in the brainstem at 4 months

**Fig. 2:**
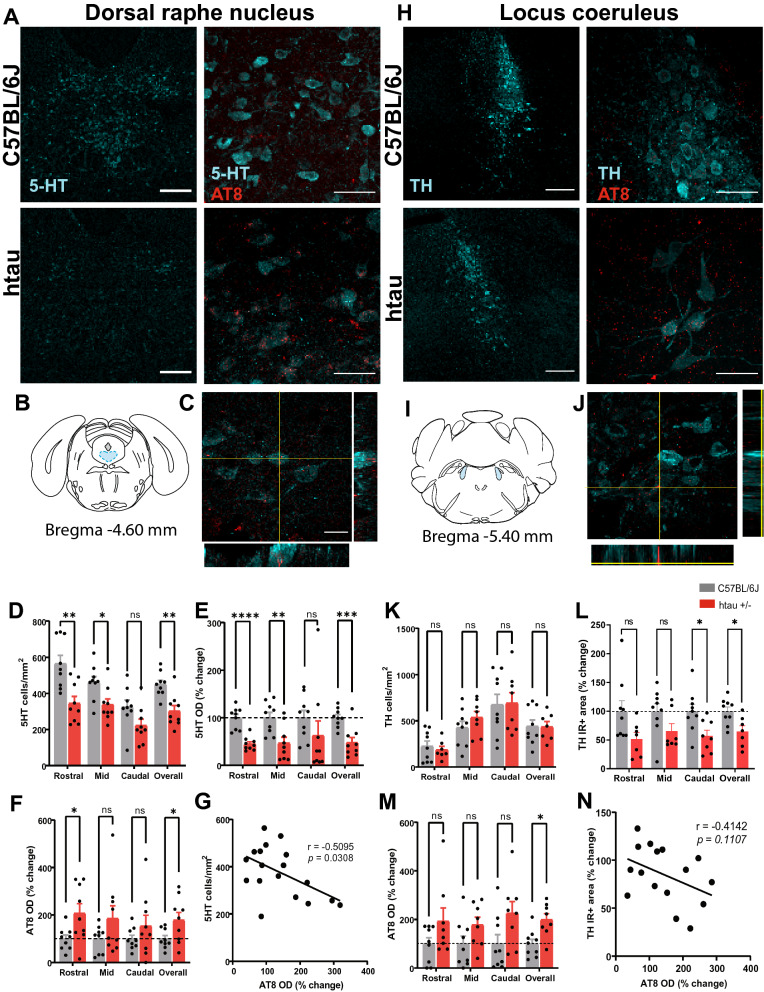
Hyperphosphorylated tau and monoaminergic depletion in the brainstem of male htau mice at 4 months. **A** Representative confocal images of 5-HT immunostaining (20X; scale bar = 200 µm) and 5-HT/AT8 co-staining (60X; scale bar = 50 µm) in the DRN of C57BL/6 J and htau +/- mice. **B** Atlas plate depicting one of the DRN regions analyzed (mid DRN; shown in blue) **C** Representative orthogonal image showing colocalization of ptau (AT8) with 5-HT neurons in the DRN (100X; scale bar = 20 µm). **D** Histogram of 5-HT cell counts/mm^2^, **E** % change in the 5-HT optical density, **F** % change in the ptau (AT8) optical density in subregions of the DRN and **G** Correlation analysis between 5-HT neuronal density and AT8 optical density in the DRN **H** Representative confocal images of TH immunostaining (20X; scale bar = 200 µm) and TH/AT8 colocalization (60X; scale bar = 50 µm) in the LC of C57BL/6 J and htau +/- mice. **I** Atlas plate depicting one of the LC regions analyzed (mid LC; shown in blue) **J** Representative orthogonal image showing colocalization of ptau with TH neurons in the LC (100X; scale bar = 20 µm). **K** Histogram of TH cell counts/mm^2^, **L** TH immunoreactive area, **M** ptau (AT8) optical density in subregions of the LC, and **N** Correlation analysis between TH immunoreactive area and AT8 optical density in the mid LC. *p < 0.05, **p < 0.01, ***p < 0.001, ****p < 0.0001

Next, we investigated whether loss of monoaminergic neurons in the DRN or LC at the 4-month mark might account for these behavioral phenotypes. We focused on males in these experiments as they had the most robust behavioral phenotypes at 4 months of age. All histological experiments were performed in separate cohorts of mice to avoid any confounds of behavioral testing on monoamine levels. The DRN was subdivided into rostral, mid and caudal subregions, which were previously found to have distinct forebrain projections and behavioral outputs [19, 45–47]. Here we found a significant reduction in the density of 5-HT neurons overall (t_16_ = 3.94, *p* < 0.01) and specifically in the rostral (t_16_ = 3.72, *p* < 0.01) and mid aspects (t_16_ = 2.75, *p* < 0.05) (Fig. 2A–D). The 5-HT optical density (OD) was also down overall (t_16_ = 4.20, *p* < 0.001) as well as in the rostral (t_16_ = 6.14, *p* < 0.0001) and mid aspects (t_16_ = 3.21, *p* < 0.01) (Fig. 2E).

We used a ptau (Ser^202^/Thr^205^) antibody (AT8) to assess tau pathology in the DRN and found a significant increase in AT8 OD overall (t_11_ = 2.26, *p* < 0.05 with Welch’s correction) specifically in the rostral portion (t_10_ = 2.49, *p* < 0.05 with Welch’s correction) (Fig. 2F). We also observed a significant negative correlation between AT8 OD and 5-HT neuronal density in the DRN (R^2^ = 0.2596, *p* < 0.05) (Fig. 2G). Orthogonal views of superimposed 5-HT and AT8 images demonstrate that there is colocalization between ptau and 5-HT neurons in DRN (Fig. 2C and Additional file 1: Fig. S2).

The LC was also subdivided into rostral, mid and caudal subregions for analysis of AT8 and TH immunoreactivity, which is the rate-limiting enzyme for norepinephrine synthesis. Surprisingly, there was no change in the density of TH neurons in any subregion of the LC (Fig. 2H–K). We did observe a reduction in TH immunoreactive area overall in the LC (t_14_ = 2.89, *p* < 0.05) and in the caudal aspect (t_15_ = 2.69, *p* < 0.05) (Fig. 2L) and an increase in AT8 OD overall (t_15_ = 3.32, *p* < 0.05) (Fig. 2M), but no significant correlation between AT8 OD and TH immunoreactive area (Fig. 2N).

It was surprising that we did not observe loss of TH-IR neurons despite the presence of hyperphosphorylated tau in this area and colocalization between phospho-tau and TH-positive neurons (Fig. 2J and Additional file 1: Fig. S2). By contrast, there was a decline in 5-HT neuronal density in the DRN that negatively correlated with AT8 immunoreactivity, suggesting that 5-HT neurons may be more susceptible to tau-induced neurodegeneration than NA neurons. The reduction in % TH immunoreactive area could also indicate that TH-positive neurons are intact but produce less TH, resulting in lower levels of norepinephrine synthesis.

As a comparison, we then examined monoaminergic neurons and ptau immunoreactivity in the DRN and LC of female mice at 4 months of age. 5-HT neuronal density was reduced overall (t_7_ = 5.27, *p* < 0.01) and more specifically in the rostral (t_7_ = 3.04, *p* < 0.05) and caudal regions (t_7_ = 3.34, *p* < 0.05) (Additional file 1: Fig. S3A–D). The 5-HT-IR area also decreased overall (t_4.40_ = 6.24, *p* < 0.01 with Welch’s correction) and in all subregions of the DRN (Rostral: t_4.34_ = 4.07, *p* < 0.05 with Welch’s correction; Mid: t_4.22_ = 4.61, *p* < 0.01 with Welch’s correction; Caudal: t_7_ = 4.42, *p* < 0.01) (Additional file 1: Fig. S3E). AT8 OD was also elevated overall (t_7_ = 2.47, *p* < 0.05) and there was a negative correlation between AT8 OD and 5-HT-IR area (R^2^ = 0.4488, *p* < 0.05) (Additional file 1: Fig. 3SF, G). Similar to what we observed in the males, there was no change in TH neuronal density in the LC (Additional file 1: Fig. S3H–K). There was a reduction in the TH-IR area in the rostral aspect (t_7_ = 6.02, *p* < 0.001) and caudal aspect (t_6_ = 11.79, *p* < 0.0001) (Additional file 1: Fig. S3L). However, there was no change in AT8 OD in any area of the LC nor was there any correlation between AT8 and TH-IR area in the LC (Additional file 1: Fig. S3 M, N).

### Glial cell activation in the brainstem of htau mice at 4 months of age

Clinical reports have suggested that neuroinflammation is a significant occurrence in AD and may play a role in the pathogenesis of the disease, promoting both tau aggregation and neurodegeneration [48, 49]. Most studies focus on the role of microglia and astrocytes which are found in abundance at sites of amyloid and tau pathology, but their function in the etiology of AD is controversial. There is some evidence of a neuroprotective role, while others have found that glial activation can promote synaptic engulfment and release cytokines that are toxic to neurons [50–53]. Mouse models of AD tend to support a neurodegenerative role of glia, and in the LC it was reported that loss of norepinephrine promotes inflammation while reducing microglia phagocytosis and clearance of β-amyloid plaques [54]. In the present study, we examined microglial and astrocytic markers Iba-1 and GFAP in the DRN and LC of 4-month-old male htau and C57BL/6J mice. There was no increase in microglial cell density in the DRN, but Iba-1 OD was elevated overall (t_8_ = 2.48, *p* < 0.05), particularly in the mid and caudal DRN aspects (t_7_ = 2.74, *p* < 0.05 and t_8_ = 2.76, *p* < 0.05, respectively) (Additional file 1: Fig. S4A–C). Additionally, there was an increase in the % change of Iba-1-IR area overall and in the caudal DRN (t_7_ = 2.70, *p* < 0.05 and t_7_ = 4.03, *p* < 0.01) (Additional file 1: Fig. S4D).

There was also no change in astrocytic cell density, but there was an increase in GFAP OD in rostral DRN (t_8_ = 3.78, *p* < 0.01), which is indicative of astrocytic activation (Additional file 1: Fig. S4E, F). There was also an increase in the % change of GFAP-IR area overall and in the caudal DRN (t_8_ = 2.38, *p* < 0.05 and t_8_ = 2.85, *p* < 0.05) (Additional file 1: Fig. S4G).

In the LC, there was a significant increase in microglial cell density overall (t_8_ = 2.82, *p* < 0.05) and in the rostral and mid LC (t_7_ = 2.43, *p* < 0.05 and t_7_ = 2.53, *p* < 0.05) (Additional file 1: Fig. S4H, I). Iba-1 OD was also elevated overall (t_8_ = 3.98, *p* < 0.05) and in all subregions of the LC (rostral: t_7_ = 4.02, *p* < 0.01; mid: t_8_ = 3.26, *p* < 0.05; caudal: t_8_ = 2.51, *p* < 0.05) (Additional file 1: Fig. S4J). Iba-1-IR area increased across the entire LC as well (t_8_ = 3.41, *p* < 0.01) and specifically in the mid LC (t_8_ = 2.69, *p* < 0.05) (Additional file 1: Fig. S4K).

In contrast to the DRN, there was no change in GFAP staining in any LC subregion (Additional file 1: Fig. S4L–N). The increase in Iba-1 in the LC is interesting in view of previous reports that microglia may migrate and become phagocytic in response to norepinephrine stimulation [54]. While we did not look at microglial migration or phagocytosis directly, it does suggest that this may be a response to the presence of tau pathology in this region. The intact TH-positive neurons in the LC at this time point may enable norepinephrine release to increase tau clearance via microglial phagocytosis.

### Reduced excitability of 5-HT neurons in the DRN of htau mice at 4 months of age

**Fig. 3:**
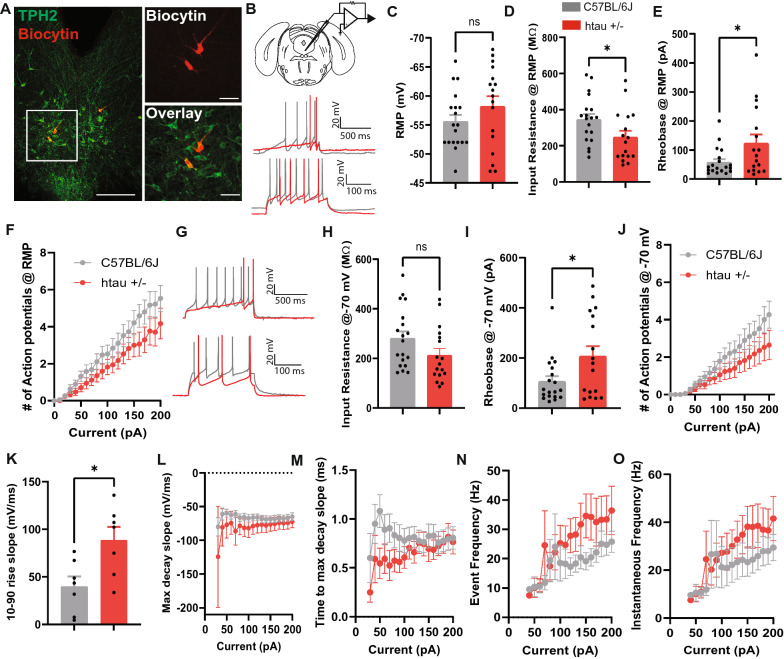
Reduction in 5-HT neuronal excitability in htau mice at 4 months. **A** Confocal images of biocytin-labeled cells (red) in the DRN overlaid with TPH2 staining (green) indicating that recorded cells were 5-HT neurons. **B** Schematic of patch clamp recordings in the DRN and representative traces of voltage ramps that were used to compute the rheobase and action potential (AP) firing at the 200 pA current step from resting membrane potential (RMP). Intracellular recordings in 5-HT neurons from 4-month-old male C57BL/6 J and htau +/- mice with histograms indicating **C** RMP, **D** Input resistance at RMP, **E** Rheobase and **F** Current (0–200 pA)-induced spiking in 250 ms from RMP **G** Representative voltage ramps and AP-current plot at 200 pA current step from a holding potential of − 70 mV. **H** Input resistance **I** Rheobase and **J** Current induced spiking (0–200 pA) from a holding potential of − 70 mV. Action potential kinetics showing the **K** 10–90% rise slope, **L** Maximum decay slope, **M** Time to maximum decay slope, **N** Event Frequency, and **O** Instantaneous frequency at 0–200 pA current steps from the RMP. *p < 0.05

The reduction in 5-HT-expressing neurons in the DRN and optical density may be indicative of neurodegeneration, which can translate to reduced neuronal activity and excitability. We used whole-cell patch clamp electrophysiology to record from 5-HT neurons in the DRN from male 4-month-old C57BL/6 J and htau mice (Fig. 3A, B). While the resting membrane potential (RMP) did not differ significantly between groups (Fig. 3C), there was a significant decrease in the input resistance (t_34_ = 2.14, *p* < 0.05) and an increase in the rheobase or AP threshold (t_34_ = 2.24, *p* < 0.05) in confirmed 5-HT neurons from htau mice that were initially at their RMP (Fig. 3D, E). This suggests that these neurons are less excitable. We also examined the firing frequency during a series of current injections from 0 to 200 pA starting at RMP, and found a main effect of current (F_20,680_ = 67.41, *p* < 0.001) and a significant interaction between genotype and current (F_20,680_ = 1.63, *p* < 0.05), suggesting that the frequency-current relationship was significantly modified in the htau mice (e.g. they fire fewer APs at higher current steps) (Fig. 3F). When the cells were held at − 70 mV, we did not observe a significant difference in input resistance, but there was a significant increase in the rheobase (t_34_ = 2.35, *p* < 0.05), a significant interaction between genotype and current injection (F_20,680_ = 1.76, *p* < 0.05), and a main effect of current (F_20,680_ = 35.32, *p* < 0.001) (Fig. 3G–J). In total, these data support the idea that 5-HT neuronal excitability is reduced in htau mice at 4 months of age.

We then examined AP kinetics and found that 5-HT neurons in htau mice have steeper rise slopes (t_12_ = 2.82, *p* < 0.05) and a higher max decay slope (genotype x current interaction: F_17,415_ = 2.84, *p* < 0.001), and the time to max decay slope was also reduced (genotype x current interaction: F_17,416_ = 1.89, *p* < 0.05) (Fig. 3K–M). This suggests faster rise and decay kinetics. There was also an increase in the event frequency with increasing stimulus intensity (F_16,328_ = 1.69, *p* < 0.05), which denotes AP frequency while the cell is firing, and in the instantaneous frequency (F_16,328_ = 2.25, *p* < 0.01), which considers the fastest instance between two APs (Fig. 3N, O). These AP kinetics are generally associated with higher excitability, but 5-HT neurons are actually less excitable.

**Fig. 4:**
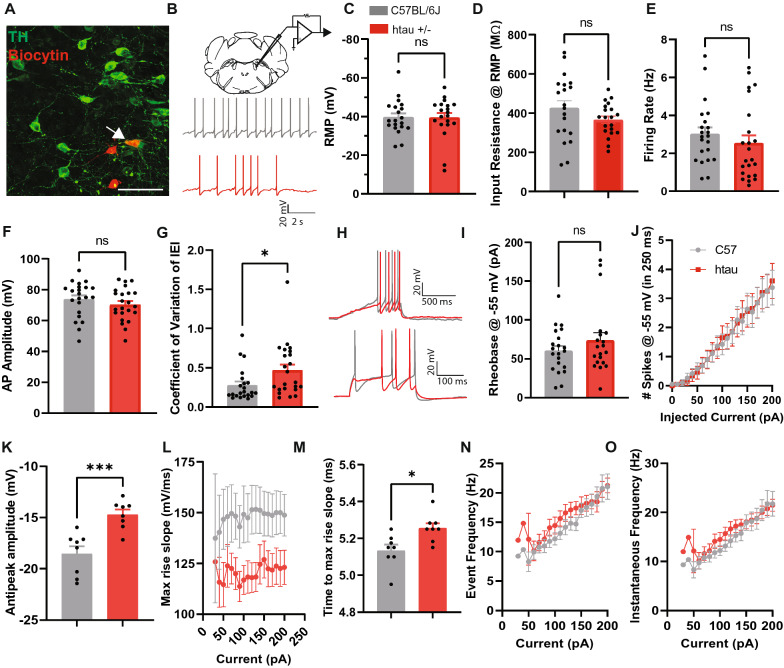
Noradrenergic neurons in the LC of htau mice display an irregular firing pattern at 4 months. **A** Confocal image of biocytin-labeled cells (red) in the LC that express TH (green) **B** Schematic of patch clamp recordings in the LC and representative traces of spontaneous action potential (AP) firing at resting membrane potential (RMP). Intracellular recordings with histograms indicating **C** RMP, **D** Input resistance at RMP, **E** Firing rate at RMP, **F** Action potential (AP) amplitude, and **G** Coefficient of variation of the inter-event interval (IEI). **H** Representative traces of voltage ramps to compute rheobase and AP-current plot at the 190 pA current step from a holding potential of − 55 mV. **I** Rheobase at − 55 mV and **J** Current (0–200 pA)-induced spiking from − 55 mV. Action potential kinetics showing the **K** Antipeak amplitude, **L** Maximum rise slope, **M** Time to maximum rise slope, **N** Event frequency, and **O** Instantaneous frequency at 0–200 pA current steps from a holding potential of − 55 mV. *p < 0.05, ***p < 0.001

Noradrenergic neurons in the LC, on the other hand, did not seem to be affected as severely affected. There was no significant change in RMP or input resistance. And since LC neurons spontaneously fired, we also measured the firing rate and AP amplitude which were found to be unaltered as well (Fig. 4A–F). There was, however, an increase in the coefficient of variation (CV) of the inter-event interval (IEI) (t_43_ = 2.26, *p* < 0.05) (Fig. 4G), suggesting that LC NA neurons fire more sporadically in htau mice. Furthermore, we measured rheobase and firing frequencies over 0–200 pA current injections from a starting potential of − 55 mV, but no group differences were observed (Fig. 4H–J). Overall, these results suggest that neuronal excitability in LC NA neurons is unchanged, although these neurons may display a more erratic firing pattern.

We also looked at AP kinetics in NA neurons and observed a decrease in antipeak amplitude (t_14_ = 4.35, *p* < 0.001), a trend toward a reduction in the max rise slope (main effect of genotype: F_1,30_ = 3.78, *p* = 0.06), and an increase in the time to max rise slope (t_14_ = 2.96, *p* < 0.05) (Fig. 4K–M). This is suggestive of slower rise kinetics coupled with smaller after hyperpolarization. However, event frequency or instantaneous frequency was unchanged (Fig. 4N, O), so overall there was no net change in electrical activity.

### Altered expression of genetic markers of monoaminergic transmission, neuroinflammation and protein aggregation in brainstem nuclei of htau mice

**Fig. 5:**
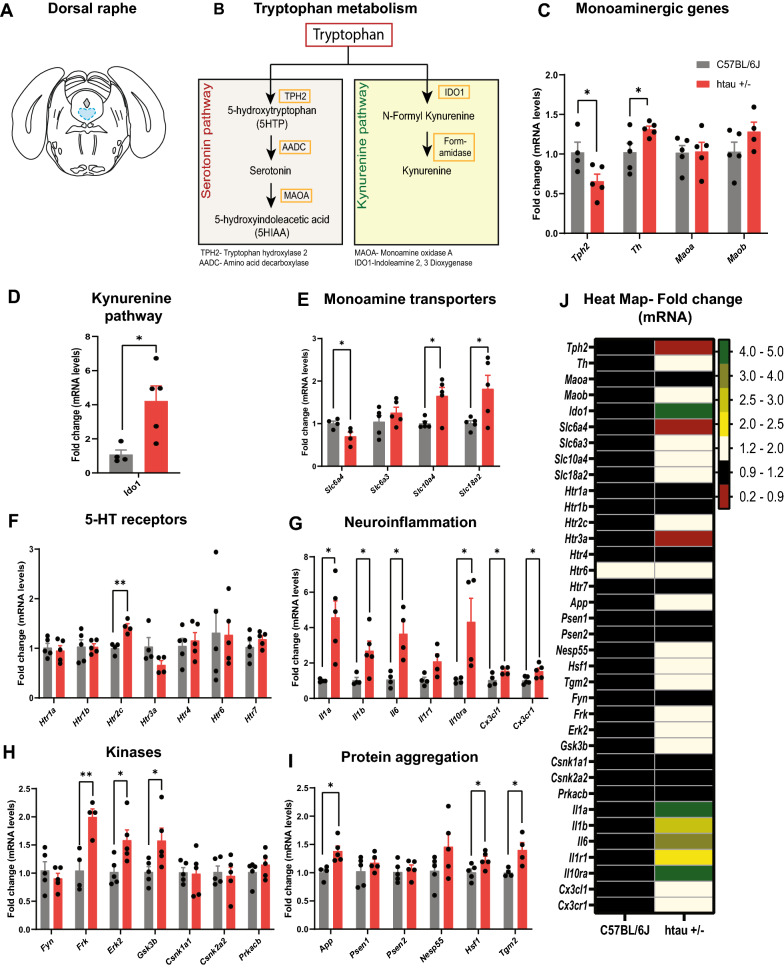
Dysregulation of gene expression in the DRN of htau +/- mice at 4 months. **A** Atlas plate indicating the DRN region that was dissected for RT-qPCR analysis. **B** Metabolic pathway for serotonin and other metabolites of tryptophan showing biosynthetic and metabolic enzymes analyzed by RT-qPCR. RT-qPCR analysis of genes involved in **C** Monoamine biosynthesis, reuptake and metabolism, **D** Kynurenine pathway, **E** Monoamine transporters, **F** 5-HT signal transduction/receptors, **G** Inflammatory pathways and **H** Protein phosphorylation and **I** Protein aggregation. **J** Heat map of gene expression for all genes analyzed. *p < 0.05, **p < 0.01

**Fig. 6:**
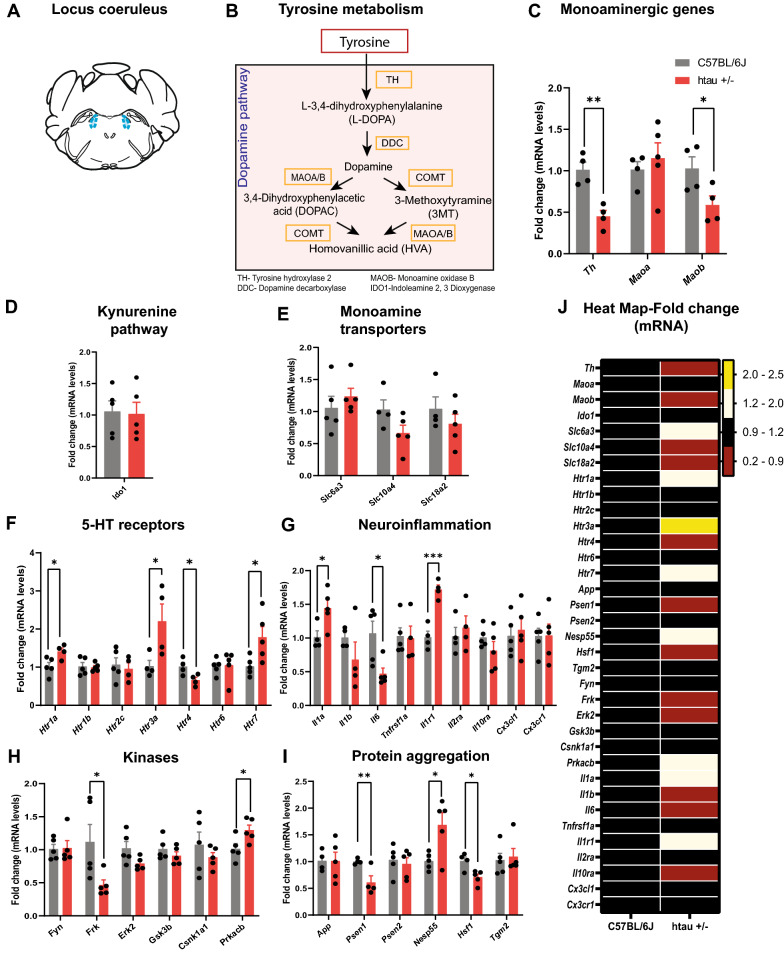
Altered gene expression in the LC of htau +/- mice at 4 months. **A** Atlas plate indicating the LC region that was dissected for RT-qPCR analysis. **B** Metabolic pathway for norepinephrine and other metabolites of tyrosine showing biosynthetic and metabolic enzymes analyzed by RT-qPCR. RT-qPCR analysis of genes involved in **C** Norepinephrine biosynthesis and metabolism **D** Kynurenine pathway **E** Monoamine transporters **F** 5-HT signal transduction/receptors, **G** Inflammatory pathways **H** Protein phosphorylation and **I** Protein aggregation. **J** Heat map of gene expression for all genes analyzed. *p < 0.05, **p < 0.01

Our results suggest that tau pathology in the DRN is associated with local neuroinflammation and altered 5-HT synthesis and neurotransmission, which may have downstream effects on cytokine production and 5-HT receptor expression in the DRN and LC. The presence of tau pathology may also be associated with upregulation of genes involved in protein phosphorylation and aggregation which could be therapeutic targets for early intervention. We next examined mRNA expression of a variety of genes involved in monoaminergic signaling and metabolism, neuroinflammation, and proteostasis in the DRN and LC of 4-month-old male htau or C57BL/6J mice (Table 3 and Figs. 5, 6). In the DRN, we found a significant reduction in *Tph2* mRNA expression (t_7_ = 2.42, *p* < 0.05; Fig. 5C), which is the rate-limiting enzyme for 5-HT synthesis. There was also an increase in tyrosine hydroxylase (*Th*: t_8_ = 2.46, *p* < 0.05) which may translate to increased dopamine synthesis (Fig. 5C). A subset of DRN neurons in the dorsal aspect of the DRN are dopaminergic and thought to play a role in rebound social behavior after periods of isolation [55]. Interestingly, there was no change in *Maoa* or *Maob* which metabolizes 5-HT to 5-HIAA (Fig. 5C), but there was an increase in *Ido1* (t_7_ = 3.03, *p* < 0.05) (Fig. 5D), the rate-limiting enzyme in the conversion of tryptophan to kynurenine. Since TPH2 competes with IDO-1 for the conversion of tryptophan to 5-HT, an increase in this enzyme together with a decrease in TPH2 would be expected to reduce 5-HT biosynthesis. We also measured the mRNA expression of monoamine transporters. Serotonin transporter (*Slc6a4* or *Sert*) mRNA was reduced as well (t_6_ = 2.69, *p* < 0.05; Fig. 5E), which is suggestive of decreased 5-HT synthesis or 5-HT neurodegeneration. *Slc6a3* which is a dopamine transporter was not altered significantly in the htau mice. However, mRNA of other monoamine transporters (*Slc10a4* and *Slc18a2*) that are involved in transporting monoamines or re-uptake mechanisms are upregulated (*Slc10a4*: t_8_ = 3.129, *p* < 0.05; *Slc18a2*: t_8_ = 2.494, *p* < 0.05) in htau mice, which is suggestive of 5-HT or TH dysfunction (Fig. 5E). No significant change in 5-HT receptor expression was observed apart from an increase in Htr_2c_ (t_6_ = 5.03, *p* < 0.01) (Fig. 5F), which may be in response to reduced 5-HTergic input to GABAergic neurons. These receptors were previously found to regulate GABAergic neurons in the DRN that provide inhibitory input to 5-HT neurons [56], so an increase in this receptor may further reduce 5-HT neuronal activity.

**Table 3:** List of primers used for quantitating mRNA expression using RT-qPCR

Gene Name	Forward/Reverse (5’-3’)	Sequence
*β-actin*	Forward	CCAGCCTTCCTTCTTGGGTA
	Reverse	GAGGTCTTTACGGATGTCAACG
*Monoaminergic transmission*		
*Slc6a4*	Forward	CAAAACGTCTGGCAAGGTGG
	Reverse	ACACCCCTGTCTCCAAGAGT
*Tph2*	Forward	GACCCAAAGACGACCTGCTT
	Reverse	CTGCGTGTAGGGGTTGAAGT
*Ido1*	Forward	GTATGTGTGGAACCGAGGGG
	Reverse	TCCAGTTTGCCAGGACACAG
*Th*	Forward	TACTTTGTGCGCTTCGAGGT
	Reverse	GGAACCTTGTCCTCTCTGGC
*Maoa*	Forward	GTATGTGAGGCAGTGTGGAGG
	Reverse	CCCCAAGGAGGACCATTATCTG
*Maob*	Forward	ATTCCACCTGCTTTGGGCAT
	Reverse	TGAACCCAAAGGCACACGA
*Slc6a3*	Forward	AAAATGGTGGAGGTGTGGGC
	Reverse	GCTAGAGTTGCTGCTATGTGC
*Slc10a4*	Forward	TCGTGAAGGTTTCCCTGTGG
	Reverse	ATGGCGACCACGTAAACAGT
*Slc18a2*	Forward	GGACCACAACTGCCCCATTA
	Reverse	CGTTAGAGGGGCTCAGTCAC
*5-HT receptors*		
*Htr1a*	Forward	TACTCCACTTTCGGCGCTTT
	Reverse	GGCTGACCATTCAGGCTCTT
*Htr1b*	Forward	ACCCTTCTTCTGGCGTCAAG
	Reverse	ACCGTGGAGTAGACCGTGT
*Htr2a*	Forward	ACCGACATGCCTCTCCATTC
	Reverse	TGACCAGTATGTTTCCCGCA
*Htr2c*	Forward	GTGCCCGTTTTTCATCACCAA
	Reverse	AGGAGGCTTTTTGTCTGGCTT
*Htr3a*	Forward	CCATCTTCATTGTGCGGCTG
	Reverse	CTTGTTGGCTTGGAAGGTGG
*Htr4*	Forward	GGAGTGTGCCAGGAGATCAG
	Reverse	AACCACTGCAAGGAACGTGA
*Htr6*	Forward	AGTGGGAGGTGGTAGGTCTC
	Reverse	GGGCTGAGGACTGATTGCTT
*Htr7*	Forward	AAGTTCTCAGGCTTCCCACG
	Reverse	TTCGCACACTCTTCCACCTC
*Neuroinflammation*		
*Il1a*	Forward	TTGCTGAAGGAGTTGCCAGA
	Reverse	GCACCCGACTTTGTTCTTTGG
*Il1b*	Forward	GCCACCTTTTGACAGTGATGAG
	Reverse	AAGGTCCACGGGAAAGACAC
*Il6*	Forward	GAGACTTCCATCCAGTTGCCT
	Reverse	TCCTCTGTGAAGTCTCCTCTCC
*Tnfrsf1a*	Forward	AGCCACACCCACAACCTTAG
	Reverse	CCCCTTAGAGACCTTTGCCC
*Il1r1*	Forward	ACTTGAGGAGGCAGTTTTCGT
	Reverse	GTCAATCTCCAGCGACAGCA
*Il1r2*	Forward	ATCTTGGTTGTGGGGGCAAT
	Reverse	CCTGGTTGTCAGTCCGTAGC
*Il2ra*	Forward	TGAAGTGTGGGAAAACGGGG
	Reverse	GCAGGAAGTCTCACTCTCGG
*Il10ra*	Forward	GCGTGACTCTGAAAGCAATGG
	Reverse	GCAGCACCTTGACACAAAACT
*Cx3cl1*	Forward	GAGAGTGAGGAAGCCAACCC
	Reverse	AAAGTCCGATGACGGGTGTC
*Cx3cr1*	Forward	GGGTTTGGTGAGTCCTGGTT
	Reverse	CAAGGAATGGACACCCGACA
*Protein aggregation*		
*App*	Forward	TTCGCTGACGGAAACCAAGA
	Reverse	TTTCGGTATTGGCTGGCACA
*Psen1*	Forward	CTCCTGCTCGCCATTTTCAAG
	Reverse	CACAAGGTAATCCGTGGCGA
*Psen2*	Forward	ACACTGAGAAGAACGGGCAG
	Reverse	AGGAGCATCAGGGAGGACAT
*Nesp55*	Forward	CCCGAGCAAGAACCTTTGGA
	Reverse	ACGGGCTCATTGTTAGACGG
*Hsf1*	Forward	AGAGGAAAGTGACCAGCGTG
	Reverse	ACAACTTTTTGCTGCTGGGC
*Gskip*	Forward	GTTCGCCATTCCTTCACACG
	Reverse	GCAGCATTGAAGCCCTGTAAC
*Tgm2*	Forward	AAGAGAAACTGGTGCTGCGT
	Reverse	GCACTGAGGCTGACCAAGAT
*Kinases*		
*Frk*	Forward	AGCAGGTCAGGAAGAAGCAC
	Reverse	CTCACCATACCTCCCGCTTC
*Fyn*	Forward	CAGCAAGACAAGGTGCGAAG
	Reverse	CCTGGGTATGGCACTCTTCC
*Erk2*	Forward	AAGACACAGCACCTCAGCAA
	Reverse	GTGTTCAGCAGGAGGTTGGA
*Gsk3b*	Forward	GGAGTGAAAAGCCAAGAGAACG
	Reverse	CAAAGGAGGTGGTTCTCGGT
*Csnk1a1*	Forward	CACAGGCAAGCAAACTGACAA
	Reverse	AACAAACGCTGCTCCAATCG
*Csnk2a2*	Forward	AAGGAGCCATTCTTCCACGG
	Reverse	TCCGTGAATGTTGTCCCAGG
*Prkacb*	Forward	CAAGAAAGGCAGCGAAGTGG
	Reverse	ATTACTCGGGGGAGGGTTCT
*Glutamatergic and Gabaergic transmission*		
*Gad1*	Forward	CCTTGAACCGTAGAGACCCC
	Reverse	TCAGGCCCAGTTTTCTGGTG
*Gad2*	Forward	ACCGTGTATGGGGCTTTTGA
	Reverse	ATCAGTAACCCTCCACCCCA
*Gls*	Forward	ACTGTAGATGGGCAAAGGCA
	Reverse	AATCCACTTGGCTCCTTCCC
*Grin1*	Forward	CTTCAGTCCCTTTGGCCGAT
	Reverse	ATGCCAGAGTTGAGCAGGAC
*Grin2a*	Forward	AGCTTGAAAACTGGGAAGTTGG
	Reverse	AGATGTACCCGCTCCCAATG
*Grin2b*	Forward	CGACCTGTACGGCAAGTTCT
	Reverse	TTGCTGCTTCCTCCTCTTGG

IDO-1 levels increase in response to inflammatory stimuli [57], and we also found that a number of pro-inflammatory genes were upregulated in htau mice including *Il-1α* (t_7_ = 3.42, *p* < 0.05), *Il-1β* (t_7_ = 2.62, *p* < 0.05), *Il-6* (t_6_ = 3.60, *p* < 0.05), *Cx3cl1* (t_6_ = 2.59, *p* < 0.05) and *Cx3cr1* (t_8_ = 2.65, *p* < 0.05) (Fig. 5G). This increased neuroinflammation may also have deleterious effects on 5-HT neurons by causing excitotoxicity [57]. There was also an increase in *Il-10r* (t_6_ = 2.49, *p* < 0.05), which may promote anti-inflammatory *Il-10* signaling to counterbalance the effects of neuroinflammation.

Dysregulation of genes that modulate tau phosphorylation was also observed (Fig. 5H). There was an increase in Fyn related Src Family Tyrosine Kinase (*Frk*; t_6_ = 4.17, *p* < 0.01), extracellular signal-regulated kinases (*Erk2*; t_8_ = 2.684, *p* < 0.05) and Glycogen synthase kinase-3 beta (*Gsk3b*; t_8_ = 2.337, *p* < 0.05) which were previously shown to promote tau phosphorylation [58, 59]. Other kinases that are known to be involved in the tau phosphorylation such as Fyn (*Fyn*), CK1/ 2 (*Csnk1a1*, *Csnk2a2*), and PKA (*Prkacb*) were not altered in our model at 4 months. We then tested mRNA expression of genes that are known to be involved in tau aggregation (Fig. 5I). TGM2, an enzyme that crosslinks proteins at lysine and glutamine residues and has been implicated in tau aggregation in AD [60–64], is upregulated in the DRN (*Tgm2*: t_6_ = 2.92, *p* < 0.05). Likewise, genes involved in protein stability including amyloid precursor protein (*App*, t_7_ = 3.28, *p* < 0.05) and heat shock factor 1 (*Hsf1*; t_8_ = 2.49, *p* < 0.05) were upregulated. HSF-1 was recently found to mediate the neuroprotective effects of 5-HT in response to stress [65, 66], so upregulation of this gene may be compensatory. Other tau aggregation-responsive genes such as neuroendocrine secretory protein 55 (*Nesp55*) and Presenilin (*Psen1* and *Psen2*) were not deregulated at this 4-month time point. All gene expression results for the DRN are also represented as a heat map in Fig. 5J.

In the LC, there was a significant reduction in *Th* expression (t_6_ = 4.87, *p* < 0.01) (Fig. 6A–C). This reduced *Th* gene expression may account for the reduction in TH optical density that we observed in the LC. Interestingly, *Maoa* which metabolizes norepinephrine is unchanged, whereas *Maob* metabolizes dopamine and is downregulated (t_6_ = 2.50, *p* < 0.05) (Fig. 6C). Moreover, in contrast to the DRN, *Ido1* and the monoamine transporter expression (*Slc6a3*, *Slc10a4*, and *Slc18a2*) were not altered in the LC (Fig. 6D and E).

We observed a significant increase in several 5-HT receptors including *Htr*_*1a*_ (t_7_ = 2.79, *p* < 0.05), *Htr*_*3a*_ (t_6_ = 2.48, *p* < 0.05), and *Htr*_*7*_ (t_8_ = 2.55, *p* < 0.05), all of which may be in response to reduced 5-HT input from the DRN (Fig. 6F). In the LC, 5-HT_3a_ receptors can stimulate local norepinephrine release in the LC which reduces firing of noradrenergic neurons, causing a reduction in distal NE release in the pre-frontal cortex (PFC) [67]. Loss of noradrenergic neurons or inhibition of their firing in the LC may play a role in the later development of anxiety-like behaviors in htau mice. In contrast, levels of *Htr*_*4*_ mRNA in the LC were reduced (t_6_ = 2.75, *p* < 0.05). 5-HT_4_ receptors were recently shown to mediate the neuroprotective effects of 5-HT on HSF-1 [65], which was also reduced (t_7_ = 3.18, *p* < 0.05) and may reflect a loss of 5-HT input to this area. We also observed an increase in pro-inflammatory genes *Il-1α* (t_7_ = 2.66, *p* < 0.05) and *Il-1r1* (t_6_ = 6.35, *p* < 0.001), although some cytokine genes like *Il-6* were downregulated (t_8_ = 3.02, *p* < 0.05) (Fig. 6G). There was also a decrease in *Frk* (t_8_ = 2.38, *p* < 0.05), and an increase in *Prkacb* gene expression (Fig. 6H). This is accompanied by the robust increase in hyperphosphorylated tau in LC. There was also a decrease in *Psen1* (t_6_ = 3.05, *p* < 0.01), which is one of the core proteins in the γ-secretase complex that generates β-amyloid from APP (Fig. 6E). While we did not directly stain for β-amyloid in this study, it has been reported that amyloid plaques are a rare occurrence in the raphe nuclei of AD patients and likely in the LC as well [13]. A heat map of all gene expression results is shown in Fig. 6J.

### Western blots confirm increased phospho-tau and monoaminergic depletion in the brainstem of htau mice

**Fig. 7:**
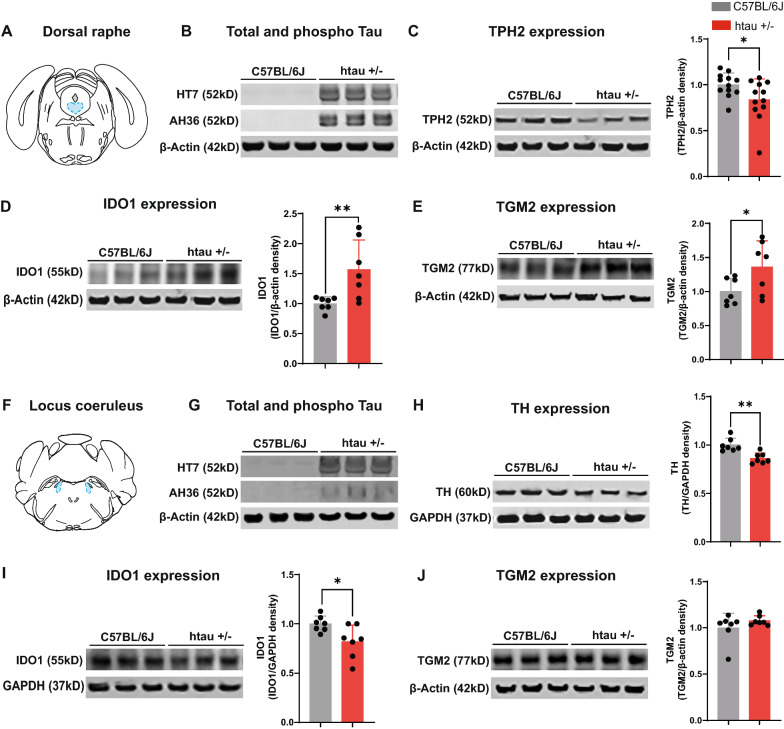
Hyperphosphorylated tau and altered expression of TPH2, TH, IDO1, and TGM2 protein levels in the monoaminergic nuclei. **A** Atlas plate indicating the DRN region that was dissected for Western blot analysis. Representative western blot images showing the **B** HT7 and AH36 **C** TPH2 **D** IDO1 **E** TGM2 protein levels in the DRN of C57BL/6 J and htau +/- . **F** Atlas plate indicating the LC region that was dissected for Western blot analysis. Representative western blot images showing the **G** HT7 and AH36 **H** TH **I** IDO1 **J** TGM2 protein levels in the LC of C57BL/6 J and htau ± . *p < 0.05, **p < 0.01

To verify the presence of hyperphosphorylated tau and monoaminergic depletion in the DRN and LC, we performed a Western blot for their markers (Fig. 7). Here we quantified TPH2 or TH expression in tissue lysates of the DRN and LC as well as ptau (Ser^202^/Thr^205^) using an antibody (AH36, StressMarq). Surprisingly, the ptau protein bands were detected with higher intensity in the DRN (Fig. 7B) as compared to LC (Fig. 7G). Moreover, the TPH2 and TH expression in the DRN and LC respectively were decreased in the htau (TPH2-t_22_ = 2.184, *p* < 0.05; TH-t_12_ = 4.163, *p* < 0.01) as compared to wild type C57BL/6J (Fig. 7C and H). Overall, the immunofluorescence and Western blot results suggest that the serotonergic system may be more susceptible to tau pathology. Moreover, we also confirmed the *Ido1* mRNA upregulation in the DRN of htau mice by an increase in the IDO-1 protein expression (t_12_ = 2.959, *p* < 0.01) (Fig. 7D). On the contrary, the IDO-1 protein expression was decreased in the LC of htau mice (t_12_ = 2.591, *p* < 0.05) (Fig. 7I). It is noted that the *Ido1* mRNA levels don’t align with the protein levels in the LC. This could be because the *Ido1* translation rate might have been altered in the htau mice. Next, we also quantified the TGM2 protein levels in the DRN and found that the TGM2 protein expression was increased in the htau mice (t_12_ = 2.205, *p* < 0.05) (Fig. 7E) and aligned with the *Tgm2* mRNA levels. On the contrary, the TGM2 protein expression was unaltered in the LC of htau as compared to C57BL/6J mice.

### Reduced serotonergic innervation and altered 5-HT receptor expression in the EC and dentate gyrus

The significant depletion of 5-HT-expressing neurons in the DRN in htau mice is expected to disrupt downstream 5-HT signaling, which may contribute to behavioral dysregulation and accelerate the progression of tau pathology in the brain. Here we observed a reduction in 5-HT immunoreactivity in all subregions (rostral, mid, caudal) of the EC at 4 months of age (Rostral: t_17_ = 2.32, *p* < 0.05; Mid: t_17_ = 2.74, *p* < 0.05; Caudal: t_17_ = 2.94, *p* < 0.01; Overall: t_17_ = 2.77, *p* < 0.05) (Additional file 1: Fig. S5A–H). We then examined serotonin transporter (SERT) immunoreactivity in the CA1, CA2, CA3 and dentate gyrus (DG) of the hippocampus, which was previously implicated in depression in an AD mouse model [27]. Surprisingly, there was no change in SERT IR area in CA1, CA2, and CA3 (CA1:t_16_ = 1.33, ns; CA2: t_16_ = 0.553, ns; t_16_ = 0.312, ns). However the SERT immunoreactivity was reduced in the DG (t_16_ = 0.174, *p* < 0.05). A previous study using viral-genetic tracing methods suggests that 5-HT projections to cortical and subcortical regions are anatomically segregated, with ventral 5-HT neurons projecting to cortical regions including the EC while dorsal 5-HT neurons project to subcortical areas [47]. This suggests that 5-HT neurons that project to the EC may be among those that are depleted at 4 months, while those that project to the hippocampus may still be intact. An alternative explanation is that both EC and hippocampal-projecting 5-HT neurons produce less 5-HT, but SERT-immunoreactive axons in the hippocampus remain intact.

We then examined gene expression in the EC and dorsal and ventral hippocampus (DHP and VHP) with a focus on genes involved in the serotonergic transmission, inflammation, and protein phosphorylation and homeostasis (summarized in Additional file 1). None of the genes involved in protein phosphorylation and homeostasis were altered in any brain region except for *Frk*, which was downregulated (EC: t_7_ = 2.44, *p* < 0.05; AHP: t_6_ = 3.27, *p* < 0.05; PHP: t_8_ = 2.51, *p* < 0.05). In the EC, there was a significant upregulation of 5-HT_2a_ receptors (t_6_ = 2.61, *p* < 0.05) which are expressed in GABAergic neurons that provide inhibitory input to principal neurons that project to the hippocampus [68]. This may be a compensatory response to reduced 5-HT input from the DRN to maintain stable network excitability in the EC. There was no change in cytokines or their receptors in the EC, although the fractalkine receptor *Cx3cr1* which is expressed in microglia is downregulated in htau mice (t_8_ = 2.54, *p* < 0.05).

Although 5-HT innervation of the hippocampus was unchanged in the CA1, CA2, and CA3, we did observe a decreased innervation in the DG region of the hippocampus. This is accompanied by an increase in *Htr*_*1a*_ mRNA expression which plays a key role in spatial learning and memory (DHP: t_7_ = 2.49, *p* < 0.05; VHP: t_8_ = 3.85, *p* < 0.01) [69]. There was also downregulation of 5-HT_1b_ receptors in the DHP (t_7_ = 4.20, *p* < 0.01), which is a presynaptic receptor that can inhibit the release of 5-HT and other neurotransmitters including glutamate. We also found an increase in *Htr*_*4*_ expression in the DHP which mediates the neuroprotective effects of 5-HT (t_6_ = 3.01, *p* < 0.05). *Htr*_*2c*_ expression was also downregulated in the DHP and the EC (DHP: t_6_ = 3.22, *p* < 0.05; EC: t_7_ = 2.78, *p* < 0.05), which is a receptor that has been implicated in anxiety-like behavior and hyperlocomotion in the dorsal (anterior) hippocampus [70]. As in the DRN and LC, cytokines and their receptors were upregulated in the DHP (*Il1r1*: t_6_ = 2.52, *p* < 0.05; *Il-1β*: t_8_ = 2.43, *p* < 0.05) and VHP (*Il-1α*: t_7_ = 2.54, *p* < 0.05; *Il-1r2*: t_8_ = 2.42, *p* < 0.05). There was also an increase in *Cx3crl1* in the DHP which can regulate microglial inflammation (t_6_ = 2.79, *p* < 0.05).

We then examined genes involved in glutamatergic and GABAergic transmission in each of these brain regions as these systems may be impacted by loss of 5-HTergic input, neuroinflammation or early neuropathological changes. None of the genes examined (*Gad1*, *Gad2*, *Gls*, *Grin1*, *Grin2a*, *Grin2b*) were modified in the EC, but we did see an upregulation of glutamatergic markers in the DHP (*Grin1*: t_7_ = 3.26, *p* < 0.05; *Grin2a*: t_7_ = 2.44, *p* < 0.05; *Grin2b*: t_7_ = 2.86, *p* < 0.05) and downregulation of GABAergic markers (*Gad2*: t_7_ = 2.71, *p* < 0.05). In the VHP, we also see a downregulation of *Gad2* (t_8_ = 2.31, *p* < 0.05) and an upregulation of the K-type mitochondrial glutaminase (*Gls*) that converts glutamine to glutamate (t_7_ = 2.37, *p* < 0.05).

## Discussion

Brainstem monoaminergic nuclei have been implicated in the early stages of AD neuropathology and may be a significant driver of prodromal neuropsychiatric symptoms. Our data in htau mice suggests that hyperphosphorylated tau appears in the DRN at an early age (4 months) and is accompanied by reductions in 5-HT immunoreactivity, neuronal excitability, and expression of serotonergic genes including *Sert* and *Tph2*. Although there was an apparent reduction in 5-HT neuronal density in the DRN, we cannot rule out the possibility that these neurons were intact, but that 5-HT expression was reduced to an undetectable level due to downregulation of *Tph2* or upregulation of *Ido-1*. These mice also displayed reduced TH immunoreactivity and lower *Th* mRNA expression in the LC without an apparent reduction in noradrenergic neuronal density. There was an overall increase in ptau immunoreactivity in the LC which may have altered *Th* gene expression in these neurons.

These monoaminergic changes were accompanied by depressive-like behaviors in htau mice, suggesting a potential link between brainstem neuropathology and depression in AD. While we cannot discern the exact brainstem locus (DRN or LC) driving the behavioral changes in this study, it is likely that both nuclei contribute to early neuropsychiatric symptoms in AD. The role of the DRN serotonergic system in mood regulation and social reward is well-documented in the literature [18, 47, 71–73], so reduced serotonergic activity may be the main driver of depressive-like behaviors in htau mice. We also found that 5-HT neurons were depleted in the rostral and mid DRN where 5-HT neurons are more ventrally localized. In a study of middle-aged patients with depression and bipolar disorders, the ventral DRN showed the largest reduction in 5-HT neurons [74], suggesting that loss of this specific 5-HT subgroup may be critical. In a recent study, 5-HT neurons in the ventral DRN were found to promote stress coping behaviors [47], so it stands to reason that loss of these neurons would result in depression. These ventral 5-HT neurons also send collateral projections to the EC, prefrontal cortex, and orbito-frontal cortex. In our study, we found a significant reduction in 5-HT innervation of the EC, and since these 5-HT neurons project to other cortical regions there is likely 5-HT denervation in the PFC and OFC as well. The EC, PFC, cingulate, insula and temporoparietal cortex have all been implicated with depression and negative affect in older adults [75, 76], so loss of 5-HT input to any one of these regions may be sufficient to drive depression in AD.

The hippocampus also receives 5-HT inputs from the DRN and has been implicated in depressive symptoms in an AD mouse model of amyloid neuropathology [27]. We did observe a reduction in *Sert* immunoreactivity in the DG which suggests that serotonergic innervation may be negatively impacted. This was accompanied by an increase in 5-HT_1A_ and 5-HT_4_ receptors which may compensate for the reduced 5-HT input. There was also a decrease in 5-HT_1B_ which could compensate for loss of 5-HT innervation by disinhibiting 5-HT release from the remaining axons. 5-HT_7_ receptors were also upregulated in the VHP and have been implicated in depressive symptoms in mice and humans [77] and may drive depressive behaviors in htau mice.

Noradrenergic neurons in the LC were relatively intact in htau mice at 4 months of age compared to 5-HT neurons. However, a few studies indicate that a minimal loss of noradrenergic neurons in the LC following a low dose of 6-hydroxydopamine (6-OHDA) can contribute to depressive-like behaviors [22, 78]. It was concluded that these depressive-like behaviors may result from the irregular firing pattern of the surviving LC neurons, which we observed in the htau mice as well. These results suggest that ptau accumulation in the LC and the resulting alterations in noradrenergic firing patterns may also contribute to depressive behaviors in htau mice. In addition, we observe anxiety-like behaviors at the 6-month mark, which may result from neuronal loss or decreased functional output of LC neurons, although we did not explicitly look for these changes at 6 months. In a recent study, induced LC neurodegeneration elevated norepinephrine (NE) turnover in downstream regions and was associated with anxiety-like behavior, suggesting that tau accumulation in the LC may have promoted anxiogenesis in htau mice [79]. Future studies targeting tau pathology in specific brain loci at various time points are needed to determine whether one or more of these brainstem nuclei contribute to specific behavioral phenotypes in htau mice.

Female htau mice exhibited fewer behavioral abnormalities than the males at 4 months but still presented with tau pathology and 5-HT depletion in the DRN, suggesting that female mice may be able to compensate for the loss of serotonergic function. This was unexpected in view of the human AD literature which asserts that females typically present with more severe neuropathology and cognitive impairments [80–83]. However, this differential rate of AD pathogenesis has been attributed to post-menopausal declines in estrogen and progesterone [84, 85], but the female mice used in this study were 4 months of age which corresponds to approximately 20–30 years in humans [86]. This suggests that sex differences may emerge in middle age when neuropathology has already taken root in the brainstem.

Hyperlocomotion in the male htau mice was also unexpected since depressive-like behaviors are usually accompanied by lower activity levels. However, both depression and overactivity are known to co-occur in AD [28, 87]. A previous study found that overexpression of IL-1β in the DRN produced “manic-like” behavior or hyperlocomotion [88], suggesting a possible link between elevated IL-1β and hyperlocomotion in htau mice. Pro-inflammatory cytokines like IL-1β activate local microglia and astrocytes that respond by releasing more cytokines and other signaling molecules, including glutamate, which can impact neuronal function and survival [51, 53, 89]. GFAP expression is elevated in the rostral DRN in proximity to areas that had the highest levels of tau accumulation, suggesting that astrocytes may contribute to protein aggregation and loss of 5-HT neurons [90, 91]. Astrocytes may also contribute to 5-HT depletion via upregulation of IDO-1, which shunts tryptophan away from 5-HT biosynthesis and toward the kynurenine pathway and oxidative stress. The apparent reduction in 5-HT-expressing neurons in the DRN of htau mice may be the result of neurodegeneration, or these neurons may produce less 5-HT but still produce other co-transmitters like glutamate that can modulate downstream cortico-striatal pathways. We observed a marked upregulation of genes involved in glutamatergic transmission in the DHP, which may be involved in novelty-induced hyperlocomotion [92]. Taken together, these data suggest that hyperlocomotion and depressive-like behaviors may arise from distinct neural pathways that are altered by pathological accumulation of tau in the DRN.

While we cannot conclusively define the anatomical origin of tau pathology in htau mice from this study, it does appear that both the DRN and LC are involved at an early stage that precedes the development of memory impairments. Tau pathology was present in both brainstem nuclei and was accompanied by neuroinflammation and depletion of monoamines. 5-HT neurodegeneration and inflammatory markers were more pronounced in the DRN, suggesting that tau pathology may develop here first or that 5-HT neurons are more sensitive to the neurotoxic effects of tau aggregation than NA neurons. We also noted that 5-HT neurons were less excitable at 4 months, while there was no change in NA neurons in the LC apart from their irregular firing pattern. The AP kinetics of 5-HT neurons were also consistent with a hyperexcitable profile even though their action potential threshold and overall firing frequency is lower. This suggests that these neurons may have been hyperexcitable at an earlier time point before they started to degenerate. This is consistent with a growing body of literature suggesting that hyperexcitability may be a precursor to neurodegeneration in AD [93, 94].

This study provides the first mechanistic evidence of tau pathology in the DRN of an AD model (htau) including monoaminergic neuronal loss, monoamine metabolite dysfunction, 5HT receptor dysregulation and behavioral impairments that are reminiscent of prodromal depression. We also demonstrate that these monoaminergic deficits are accompanied by increased neuroinflammation and altered expression of genes that promote tau phosphorylation and aggregation including *Frk* and *Tgm2*, which may be targeted for therapeutic intervention. These results strongly suggest that the htau mouse is a viable model of prodromal AD that lends itself to delineating the mechanisms driving early tau accumulation in monoaminergic neurons and their role in the progression of AD. The co-occurrence of depressive behavior, tau pathology, and 5-HT dysfunction in these mice also support a role for DRN neuropathology and the attendant 5-HT neurodegeneration in these early symptoms, although further studies are still needed to establish a causal relationship. This initial investigation lays the foundation for such a study and suggests several targets (e.g. *Tph2*, *Frk*, *Tgm2*) that could be used to rescue depressive-like behaviors in these mice. Loss of 5-HT innervation in the EC and DG region of the hippocampus also suggests that specific subgroups of 5-HT neurons that promote stress-coping behaviors may be more vulnerable to neurodegeneration. This study also adds to a growing body of literature that neuropathology develops in the brainstem in the early stages of AD and may also promote non-cognitive symptoms. Further studies are needed to define the role of DRN and LC neurons in specific behavioral symptoms in htau mice.

## Conclusion

Tau accumulation in the DRN and subsequent loss of monoaminergic drive may promote depressive-like behaviors in the prodromal phase of AD prior to the onset of cognitive decline. Further studies in AD mouse models are needed to define a causal relationship between DRN or LC neuropathology and specific AD symptoms.

## Supplementary Information

**Additional file 1: Supplementary Methods**: RT-PCR quantification and tau splice isoforms; Detection of tau isoforms in whole brain lysates. **Supplementary Figure 1**: Validation of htau mice showing presence of insoluble 3R tau isoform in whole brain lysates at 12 months of age. (A) Mouse and human tau splice isoforms in C57BL/6J, htau +/- and MAPT -/- mice. The human-specific primers only amplified products in htau +/- while mouse-specific primers only amplified products in C57BL/6J mice. (B) (i) Protein expression of human tau (HT7) in total protein extracts and (ii) 3R and 4R tau isoforms in Sarkosyl soluble and insoluble fractions from C57BL/6J, htau +/- and MAPT -/- mice. Only htau +/- mice contained human-specific tau (HT7) and the 3R tau isoform in detergent insoluble fractions. Both htau +/- and C57 mice contained the 4R isoform in soluble fractions. **Supplementary Figure 2**: Orthogonal images confirming hyperphosphorylated tau in 5-HT neurons in the DRN. (A) Atlas plate depicting the mid region of the DRN that was used to obtain orthogonal planes. (B-D) Representative orthogonal planes showing colocalization between 5-HT (cyan) and ptau (red) in the DRN. (E) Atlas plate depicting the mid region of the LC that was used to obtain orthogonal planes. (F-H) Representative orthogonal planes showing colocalization between TH (cyan) and ptau (red) in the LC. **Supplementary Figure 3**: Hyperphosphorylated tau and monoaminergic depletion in the brainstem of female htau mice at 4 months. (A) Representative confocal images of 5-HT immunostaining (20X; scale bar = 200 µm) and 5-HT/AT8 co-staining (60X; scale bar = 50 µm) in the DRN of C57BL/6J and htau +/- mice. (B) Atlas plate depicting one of the DRN regions analyzed (mid DRN) (C) Representative orthogonal image showing colocalizaton of ptau with 5-HT neurons in the DRN (100X; scale bar = 20 µm). (D) Histogram of 5-HT cell counts/mm2, (E) 5-HT immunoreactive area (%), (F) ptau (AT8) optical density in subregions of the DRN and (G) Correlation analysis between 5-HT IR area and AT8 optical density in the DRN (H) Representative confocal images of TH immunostaining (20X; scale bar = 200 µm) and TH/AT8 colocalization (60X; scale bar = 50 µm) in the LC of C57BL/6J and htau +/- mice. (I) Atlas plate depicting one of the LC regions analyzed (mid LC) (J) Representative orthogonal image showing colocalization of ptau with TH neurons in the LC (100X; scale bar = 20 µm). (K) Histogram of TH cell counts/mm2, (L) TH immunoreactive area, (M) ptau (AT8) optical density in subregions of the LC, and (N) Correlation analysis between TH immunoreactive area and AT8 optical density in the mid LC. *p < 0.05, **p < 0.01, ***p < 0.001. **Supplementary Figure 4**: Glial activation in the DRN and LC in htau mice at 4 months. (A) Representative confocal images of Iba-1 and GFAP immunostaining in the DRN of C57 and htau +/- mice (60X; scale bar = 50 µm). (B) Iba-1+ cell counts/mm2, (C) Iba-1 optical density, and (D) Iba-1 immunoreactive area in subregions of the DRN. (E) GFAP+ cells/mm2, (F) GFAP optical density, and (G) GFAP immunoreactive area in subregions of the DRN. (H) Representative confocal images of Iba-1 and GFAP immunostaining in the LC of C57 and htau +/- mice. (I) Iba-1+ cells/mm2, (J) Iba-1 optical density, and (K) Iba-1 immunoreactive area in subregions of the LC. (L) GFAP+ cells/mm2, (M) GFAP optical density, and (N) GFAP immunoreactive area in subregions of the LC. Data are represented as mean ± SEM. *p < 0.05, **p < 0.01. White arrows denote Iba-1 +or GFAP+ cell bodies. **Supplementary Figure 5**: Reduced 5-HT innervation of the entorhinal cortex in htau mice at 4 months. Representative confocal images of 5-HT fiber immunostaining (40x; scale bar = 50 um) in C57 and htau mice. (A) Atlas representation of a section of the EC used for analysis (in red). (B) Representative confocal images of 5-HT staining in the EC of C57BL/6J and htau mice at 40X. (C) Histogram of % 5-HT IR area in subregions of the EC. (D) Representative confocal images of 5-HT staining in the EC at 100X. (E) Atlas representation of CA1 region of the hippocampus used in the analysis (in red). (F) Representative confocal image of SERT staining in the CA1 region of the hippocampus in C57BL/6J and htau mice. (G) Histogram of % SERT IR area in subregions of the hippocampus (H) Representative confocal images of SERT staining in the CA1 region of the hippocampus at 100X. Data are represented as mean ± SEM. *p < 0.05, **p < 0.01. **Supplementary Table 1**: List of fold change values obtained from RT-qPCR of genes tested in the dorsal raphe nucleus, locus coeruleus, dorsal and ventral hippocampus, and entorhinal cortex of C57 and htau (*p < 0.05, **p < 0.01, ***p < 0.001).

## Data Availability

All data generated or analyzed during this study are included in this published article and its Additional file 1.
