# Supplementary material for: Human tau-overexpressing mice recapitulate brainstem involvement and neuropsychiatric features of early Alzheimer’s disease
Source: Acta Neuropathol Commun. 2023 Apr 3;11:57. doi: 10.1186/s40478-023-01546-5 (PMC10069039; doi:10.1186/s40478-023-01546-5)
Supplement: Supplementary file 1 — Additional file 1: Supplementary Methods: RT-PCR quantification and tau splice isoforms; Detection of tau isoforms in whole brain lysates. Supplementary Figure 1: Validation of htau mice showing presence of insoluble 3R tau isoform in whole brain lysates at 12 months of age. (A) Mouse and human tau splice isoforms in C57BL/6J, htau +/- and MAPT -/- mice. The human-specific primers only amplified products in htau +/- while mouse-specific primers only amplified products in C57BL/6J mice. (B) (i) Protein expression of human tau (HT7) in total protein extracts and (ii) 3R and 4R tau isoforms in Sarkosyl soluble and insoluble fractions from C57BL/6J, htau +/- and MAPT -/- mice. Only htau +/- mice contained human-specific tau (HT7) and the 3R tau isoform in detergent insoluble fractions. Both htau +/- and C57 mice contained the 4R isoform in soluble fractions. Supplementary Figure 2: Orthogonal images confirming hyperphosphorylated tau in 5-HT neurons in the DRN. (A) Atlas plate depicting the mid region of the DRN that was used to obtain orthogonal planes. (B-D) Representative orthogonal planes showing colocalization between 5-HT (cyan) and ptau (red) in the DRN. (E) Atlas plate depicting the mid region of the LC that was used to obtain orthogonal planes. (F-H) Representative orthogonal planes showing colocalization between TH (cyan) and ptau (red) in the LC. Supplementary Figure 3: Hyperphosphorylated tau and monoaminergic depletion in the brainstem of female htau mice at 4 months. (A) Representative confocal images of 5-HT immunostaining (20X; scale bar = 200 µm) and 5-HT/AT8 co-staining (60X; scale bar = 50 µm) in the DRN of C57BL/6J and htau +/- mice. (B) Atlas plate depicting one of the DRN regions analyzed (mid DRN) (C) Representative orthogonal image showing colocalizaton of ptau with 5-HT neurons in the DRN (100X; scale bar = 20 µm). (D) Histogram of 5-HT cell counts/mm2, (E) 5-HT immunoreactive area (%), (F) ptau (AT8) optical density in subregions of the DRN and [file 40478_2023_1546_MOESM1_ESM.docx]

**SUPPLEMENTARY METHODS**

***RT-PCR quantification of tau splice isoforms***

To examine the 3R (3 repeats; 293bp) and 4R (4 repeats; 390bp) *MAPT* isoform, we performed RT-PCR using specific primers reported previously [30,31]. Briefly, total RNA was extracted from cortical tissues of WT, htau +/- and htau -/- mice brains using TRIzol reagent (Ambion, Life Technologies, USA) as described previously [29]. The total RNA was reverse transcribed using the iScript™ cDNA Synthesis kit (Bio-Rad Laboratories, CA, USA) with a thermal profile: 25 °C for 5 min, 45 °C for 20 min, and 95 °C for 1 min. Following this, RT-PCR was performed using the thermal profile: 95 °C for 3 min followed by 40 cycles of 95 °C for 30 s, 60 °C for 30 s, and 72 °C for 1 min and final extension at 72 °C for 5 mins. After RT-PCR, the products were resolved in a 2% agarose gel and imaged under UVPTM gel imaging system (UVP LLC, CA, USA).
***Detection of tau isoforms in whole brain lysates***

For detection of total human tau, tissues were weighed and homogenized in RIPA buffer at 150 µl/mg tissue using a sonicator. For detection 3R and 4R isoforms, tissues were homogenized in high-salt (HS) lysis buffer using a bead mill homogenizer [33–38]. The homogenate was then centrifuged at 130,000 *x g* for 30 minutes at 4⁰C. The supernatant was removed, and the tissue pellet was resuspended in 1% Triton X-100 in HS buffer and centrifuged at 130,000 *x g* for 30 minutes at 4⁰C. The supernatant was removed, and this process repeated a second time. To isolate the soluble and insoluble fraction, the pellet was resuspended in 1% of Sarkosyl was in HS buffer and incubated for 30 minutes in a water bath at 37⁰C. This suspension was then centrifuged at 130,000 *x g* for 30 mins at 4°C. The supernatant was removed (Sarkosyl soluble fraction), and the pellet re-suspended in urea buffer (0.5 ml of 4 M urea, 2% SDS, and 25 mM Tris-HCl, pH 7.6; Sarkosyl insoluble fraction). Protein concentration was measured using the Pierce™ 660nm Protein Assay Kit (ThermoFisher Scientifc) following the manufacturer’s protocol.

Extracted protein lysate samples were mixed with Laemmli buffer (Bio-Rad Laboratories, USA) and denatured at 95°C for 5 mins. Samples were then loaded into a precast 10-well 10% TGX SDS-PAGE gel (BioRad Laboratories, USA), and proteins were transferred to a PVDF membrane by using semi-dry Trans-Blot Turbo Transfer System (BioRad Laboratories, USA). Then the membranes were transferred into blocking solution (10% BSA or skimmed milk powder in PBS with 0.1% Tween20). Membranes were probed with primary antibodies as follows: HT7 (Total human tau; 1:3000), RD3 (3R; 1:200), and RD4 (4R; 1:200). HT7 was obtained from ThermoFisher and RD3 and RD4 were generous gifts from Gloria Lee. The following day, all membranes were probed with appropriate secondary antibodies (IRDye 680RD; 1:2000 or IRDye 800CW; 1: 3000; Li-Cor Biotechnology, USA) for 60 mins followed by 3 washes with PBS-T. Membranes were then imaged with a Sapphire™ Biomolecular Imager (Azure Biosystems, Inc).

**SUPPLEMENTARY FIGURES**

**
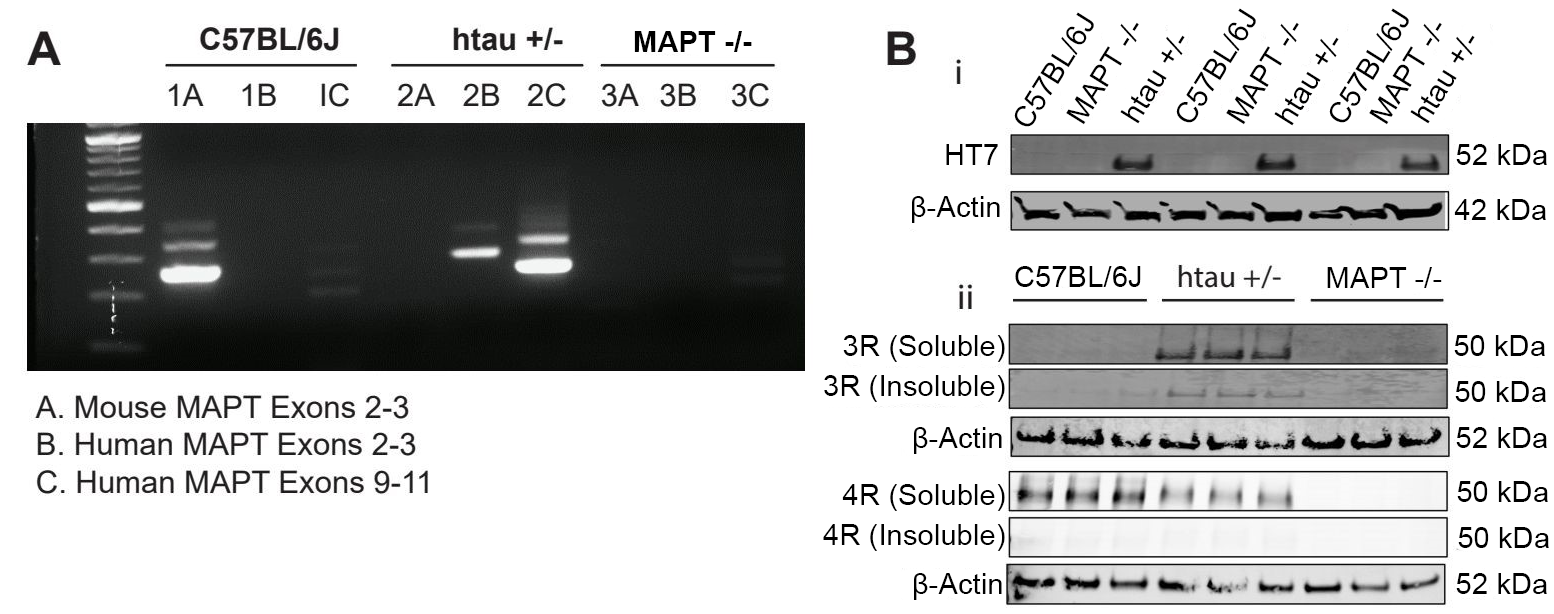
**

**Supplementary Figure 1: Validation of htau mice showing presence of insoluble 3R tau isoform in whole brain lysates at 12 months of age.** (A) Mouse and human tau splice isoforms in C57BL/6J, htau +/- and *MAPT* -/- mice. The human-specific primers only amplified products in htau +/- while mouse-specific primers only amplified products in C57BL/6J mice. (B) (i) Protein expression of human tau (HT7) in total protein extracts and (ii) 3R and 4R tau isoforms in Sarkosyl soluble and insoluble fractions from C57BL/6J, htau +/- and *MAPT* -/- mice. Only htau +/- mice contained human-specific tau (HT7) and the 3R tau isoform in detergent insoluble fractions. Both htau +/- and C57 mice contained the 4R isoform in soluble fractions.


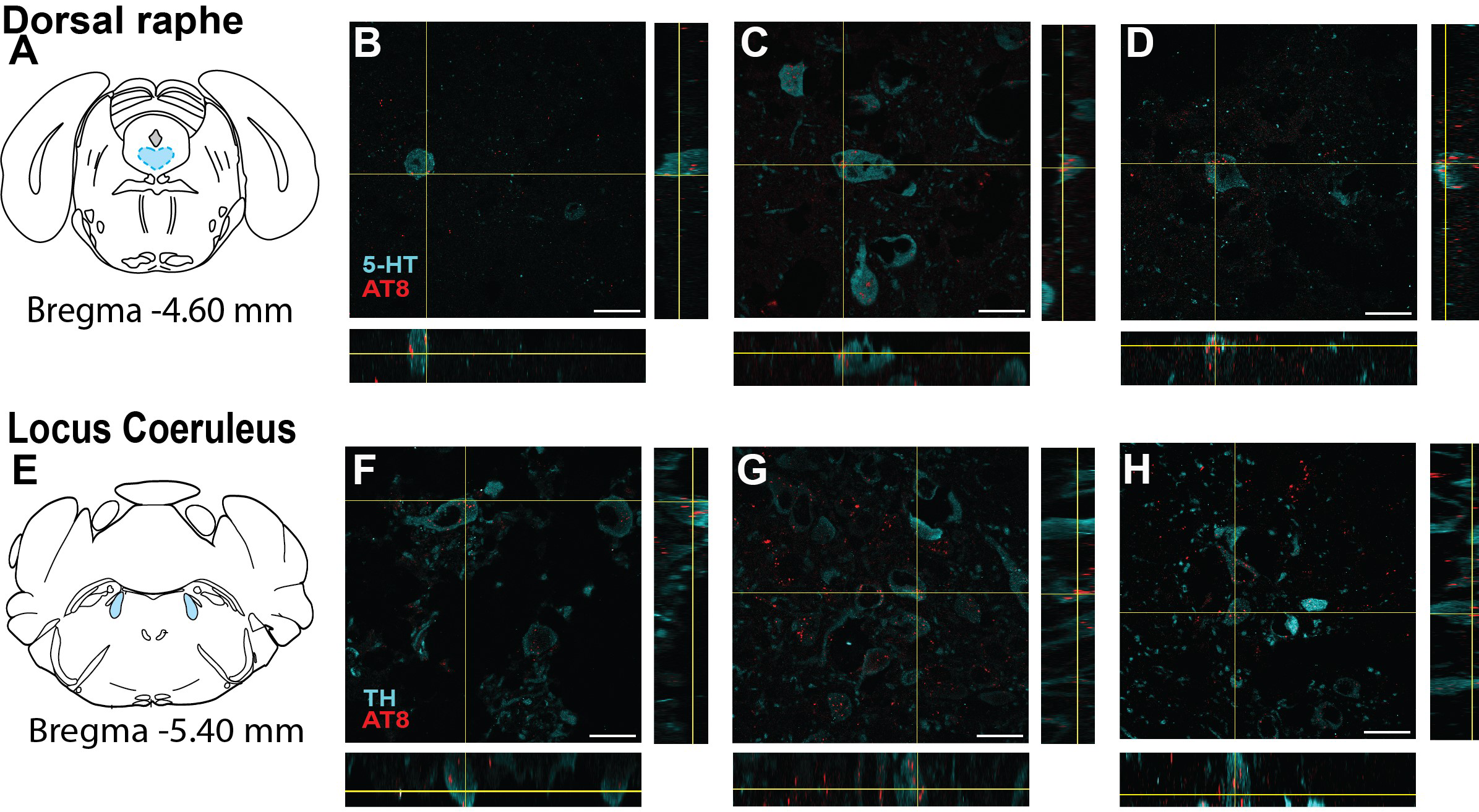


**Supplementary Figure 2: Orthogonal images confirming hyperphosphorylated tau in 5-HT neurons in the DRN.** (A) Atlas plate depicting the mid region of the DRN that was used to obtain orthogonal planes. (B-D) Representative orthogonal planes showing colocalization between 5-HT (cyan) and ptau (red) in the DRN. (E) Atlas plate depicting the mid region of the LC that was used to obtain orthogonal planes. (F-H) Representative orthogonal planes showing colocalization between TH (cyan) and ptau (red) in the LC.

**
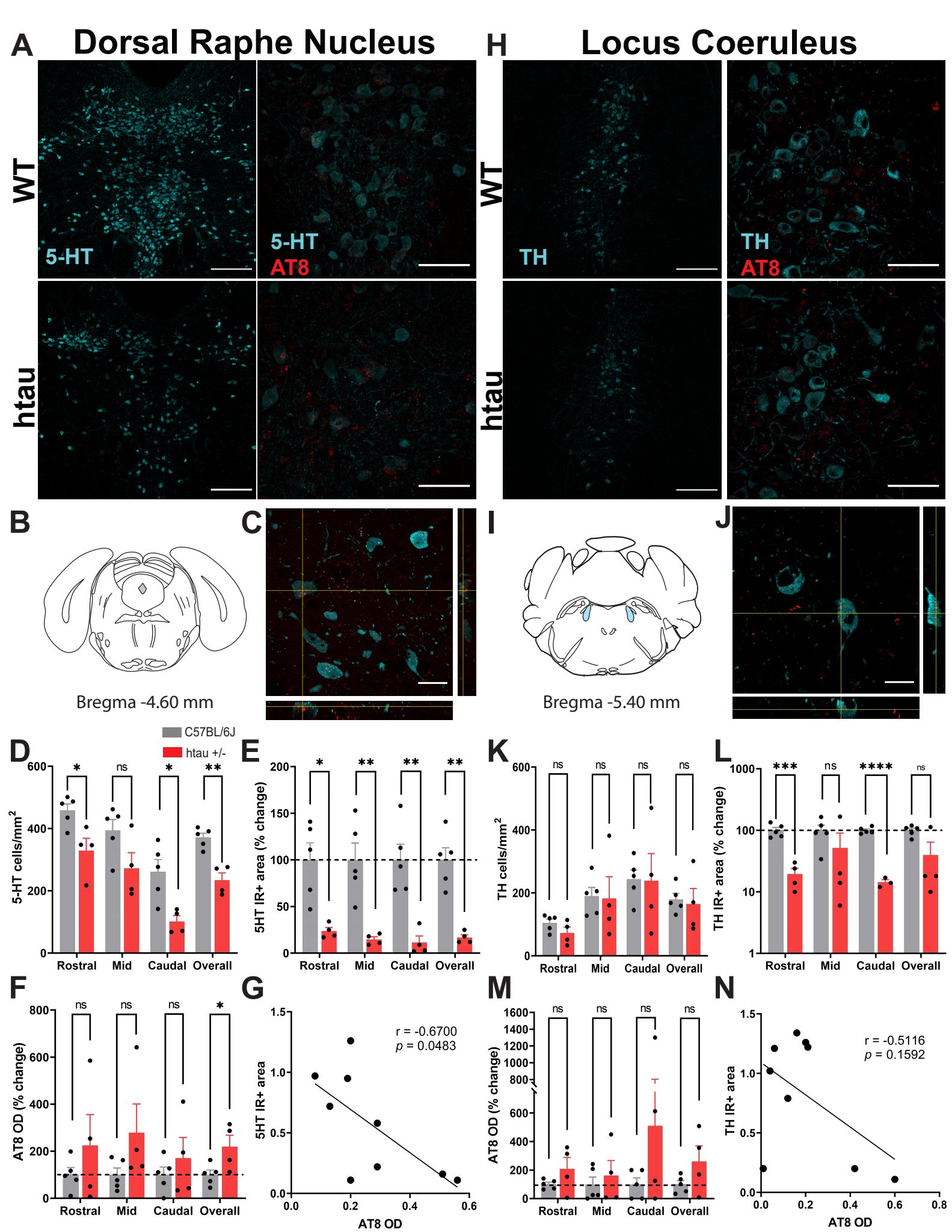
Supplementary Figure 3: Hyperphosphorylated tau and monoaminergic depletion in the brainstem of female htau mice at 4 months.** (A) Representative confocal images of 5-HT immunostaining (20X; scale bar = 200 µm) and 5-HT/AT8 co-staining (60X; scale bar = 50 µm) in the DRN of C57BL/6J and htau +/- mice. (B) Atlas plate depicting one of the DRN regions analyzed (mid DRN) (C) Representative orthogonal image showing colocalizaton of ptau with 5-HT neurons in the DRN (100X; scale bar = 20 µm). (D) Histogram of 5-HT cell counts/mm^2^, (E) 5-HT immunoreactive area (%), (F) ptau (AT8) optical density in subregions of the DRN and (G) Correlation analysis between 5-HT IR area and AT8 optical density in the DRN (H) Representative confocal images of TH immunostaining (20X; scale bar = 200 µm) and TH/AT8 colocalization (60X; scale bar = 50 µm) in the LC of C57BL/6J and htau +/- mice. (I) Atlas plate depicting one of the LC regions analyzed (mid LC) (J) Representative orthogonal image showing colocalization of ptau with TH neurons in the LC (100X; scale bar = 20 µm). (K) Histogram of TH cell counts/mm^2^, (L) TH immunoreactive area, (M) ptau (AT8) optical density in subregions of the LC, and (N) Correlation analysis between TH immunoreactive area and AT8 optical density in the mid LC. *p<0.05, **p<0.01, ***p<0.001.

**
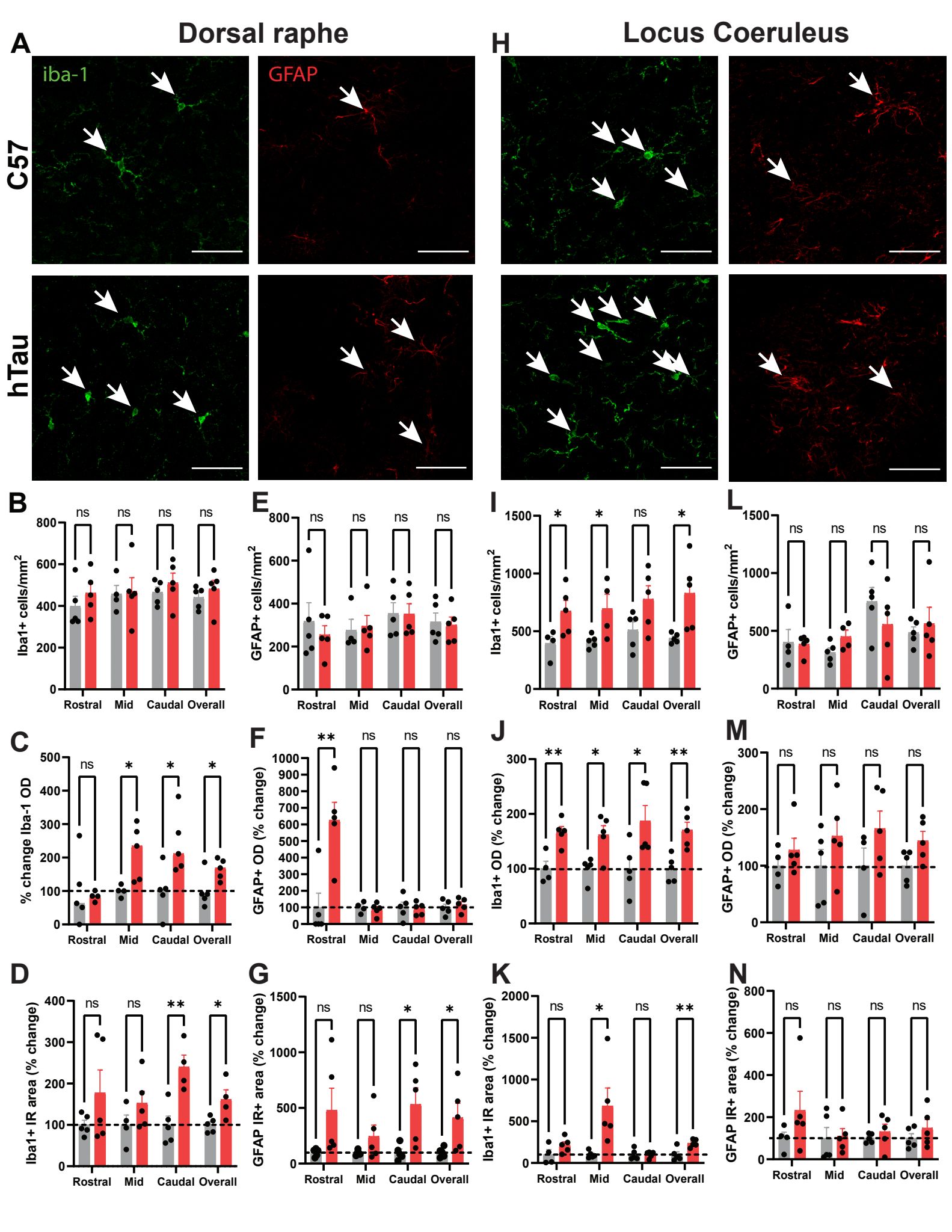
**

**Supplementary Figure 4: Glial activation in the DRN and LC in htau mice at 4 months.** (A) Representative confocal images of Iba-1 and GFAP immunostaining in the DRN of C57 and htau +/- mice (60X; scale bar = 50 µm). (B) Iba-1+ cell counts/mm^2^, (C) Iba-1 optical density, and (D) Iba-1 immunoreactive area in subregions of the DRN. (E) GFAP+ cells/mm^2^, (F) GFAP optical density, and (G) GFAP immunoreactive area in subregions of the DRN. (H) Representative confocal images of Iba-1 and GFAP immunostaining in the LC of C57 and htau +/- mice. (I) Iba-1+ cells/mm2, (J) Iba-1 optical density, and (K) Iba-1 immunoreactive area in subregions of the LC. (L) GFAP+ cells/mm^2^, (M) GFAP optical density, and (N) GFAP immunoreactive area in subregions of the LC. Data are represented as mean ± SEM. *p<0.05, **p<0.01. White arrows denote Iba-1 +or GFAP+ cell bodies.


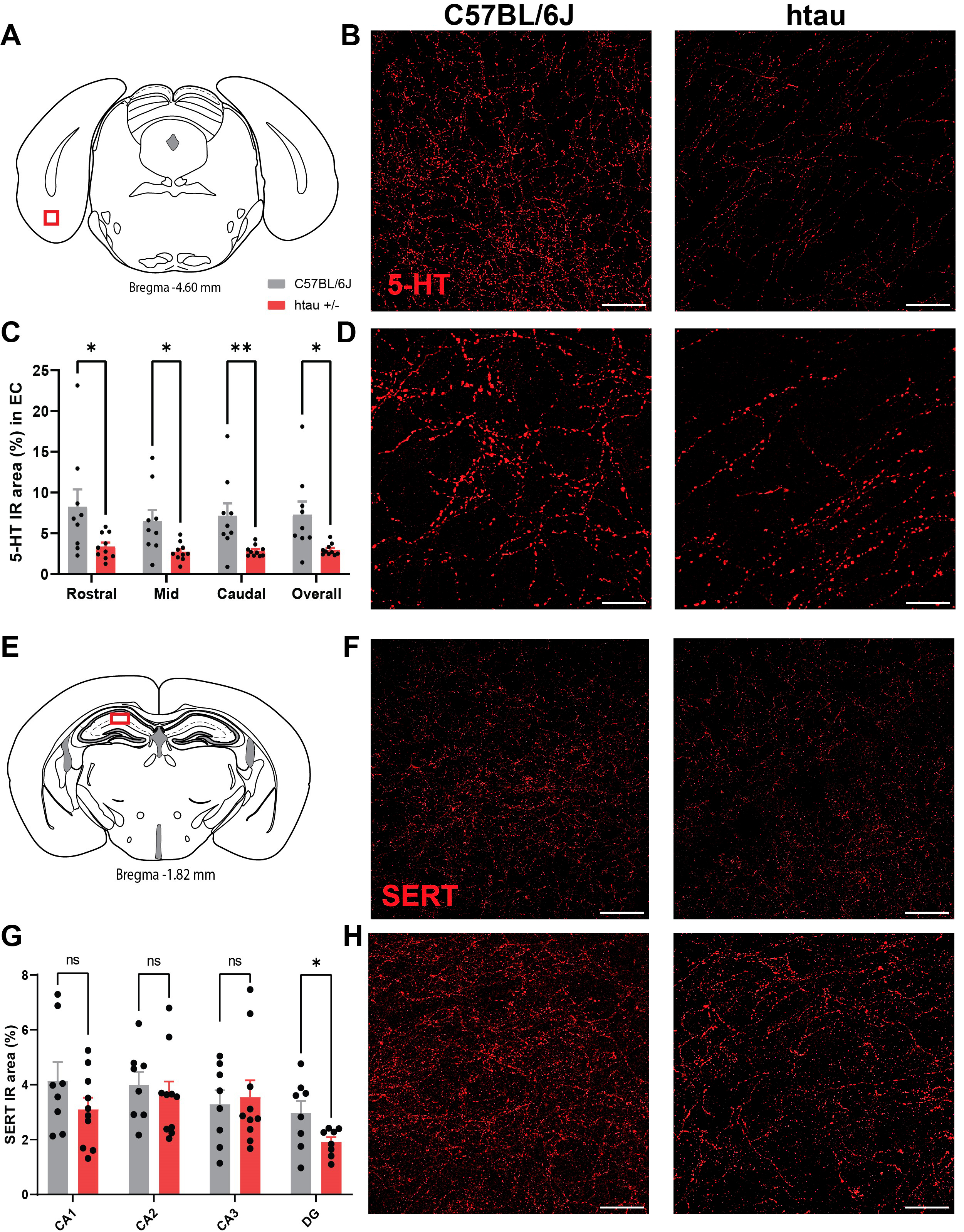


**Supplementary Figure 5: Reduced 5-HT innervation of the entorhinal cortex in htau mice at 4 months.** Representative confocal images of 5-HT fiber immunostaining (40x; scale bar = 50 um) in C57 and htau mice. (A) Atlas representation of a section of the EC used for analysis (in red). (B) Representative confocal images of 5-HT staining in the EC of C57BL/6J and htau mice at 40X. (C) Histogram of % 5-HT IR area in subregions of the EC. (D) Representative confocal images of 5-HT staining in the EC at 100X. (E) Atlas representation of CA1 region of the hippocampus used in the analysis (in red). (F) Representative confocal image of SERT staining in the CA1 region of the hippocampus in C57BL/6J and htau mice. (G) Histogram of % SERT IR area in subregions of the hippocampus (H) Representative confocal images of SERT staining in the CA1 region of the hippocampus at 100X. Data are represented as mean ± SEM. *p<0.05, **p<0.01.

**Supplementary table 1: List of fold change values obtained from RT-qPCR of genes tested in the dorsal raphe nucleus, locus coeruleus, dorsal and ventral hippocampus, and entorhinal cortex of C57 and htau (*p<0.05; **p<0.01; ***p<0.001).**

N/A: not tested; BDL: Below detection limit

|  |  | **Fold Change** | | | | |
| --- | --- | --- | --- | --- | --- | --- |
|  | **Gene Name** | **Dorsal raphe nucleus** | **Locus coeruleus** | **Dorsal hippocampus**  **(DHP)** | **Ventral hippocampus**  **(VHP)** | **Entorhinal cortex** |
| ***Monoaminergic transmission*** | *Slc6a4* | 0.70* | N/A | N/A | N/A | N/A |
|  | *Tph2* | 0.65* | N/A | N/A | N/A | N/A |
|  | *Ido1* | 4.21* | 1.01 | N/A | N/A | N/A |
|  | *Th* | 1.31* | 0.44** | N/A | N/A | N/A |
|  | *Maoa* | 1.03 | 1.15 | N/A | N/A | N/A |
|  | *Maob* | 1.28 | 0.58* | N/A | N/A | N/A |
|  | *Slc6a3* | 1.26 | 1.30 | N/A | N/A | N/A |
|  | *Slc10a4* | 1.65* | 0.66 | N/A | N/A | N/A |
|  | *Slc18a2* | 1.82* | 0.81 | N/A | N/A | N/A |
| ***5-HT receptors*** | *Htr1a* | 0.95 | 1.41* | 2.45* | 1.57** | 0.91 |
|  | *Htr1b* | 1.03 | 0.99 | 1.05 | 0.61** | 0.92 |
|  | *Htr2a* | N/A | N/A | 1.06 | 1.13 | 1.72* |
|  | *Htr2c* | 1.43* | 0.95 | 0.39* | 0.91 | 0.35* |
|  | *Htr3a* | 0.66 | 2.20* | BDL | BDL | BDL |
|  | *Htr4* | 1.16 | 0.66* | 2.38* | 1.04 | 2.12 |
|  | *Htr6* | 1.27 | 1.05 | 0.94 | 1.14 | 1.06 |
|  | *Htr7* | 1.18 | 1.78* | 1.00 | 1.43* | 0.84* |
| ***Neuroinflammation*** | *Il1a* | 4.58* | 1.44* | 0.86 | 0.75* | 1.21 |
|  | *Il1b* | 2.69* | 0.67 | 0.39* | 0.71 | 1.32 |
|  | *Il6* | 3.65* | 0.46* | BDL | BDL | BDL |
|  | *Tnfrsf1a* | N/A | 0.99 | N/A | N/A | N/A |
|  | *Il1r1* | 2.09 | 1.71*** | 0.64* | 0.84 | 0.87 |
|  | *Il1r2* | N/A | N/A | 1.18 | 1.59* | 1.20 |
|  | *Il2ra* | BDL | 1.15 | N/A | N/A | N/A |
|  | *Il10ra* | 4.32* | 0.81 | 1.08 | 0.93 | 1.07 |
|  | *Cx3cl1* | 1.55* | 1.12 | 1.35* | 1.04 | 0.92 |
|  | *Cx3cr1* | 1.53* | 1.03 | 0.92 | 0.85 | 0.84* |
| ***Protein aggregation*** | *App* | 1.38* | 1.01 | 1.03 | 1.01 | 0.92 |
|  | *Psen1* | 1.17 | 0.60* | 0.97 | 0.89 | 1.06 |
|  | *Psen2* | 1.06 | 0.95 | 0.98 | 1.02 | 0.92 |
|  | *Nesp55* | 1.46 | 1.69* | N/A | N/A | N/A |
|  | *Hsf1* | 1.22* | 0.70* | 1.08 | 1.02 | 1.08 |
|  | *Gskip* | N/A | N/A | 0.90 | 1.08 | 0.91 |
|  | *Tgm2* | 1.40* | 1.09 | 1.06 | 0.94 | 0.88 |
|  | *Frk* | 1.99** | 0.46* | 0.54* | 0.69* | 0.75* |
|  | *Fyn* | 0.91 | 1.03 | N/A | N/A | N/A |
|  | *Erk2* | 1.58* | 0.79 | N/A | N/A | N/A |
|  | *Gsk3b* | 1.58* | 0.91 | N/A | N/A | N/A |
|  | *Csnk1a1* | 0.99 | 0.88 | N/A | N/A | N/A |
|  | *Csnk2a2* | 0.95 | N/A | N/A | N/A | N/A |
|  | *Prkacb* | 1.15 | 1.29* | N/A | N/A | N/A |
| ***Glutamatergic/Gabaergic markers*** | *Gad1* | N/A | N/A | 0.88 | 0.99 | 1.02 |
|  | *Gad2* | N/A | N/A | 0.64* | 0.76* | 1.03 |
|  | *Gls* | N/A | N/A | 1.01 | 1.32* | 1.12 |
|  | *Grin1* | N/A | N/A | 1.28* | 1.13 | 1.06 |
|  | *Grin2a* | N/A | N/A | 1.54* | 0.96 | 1.07 |
|  | *Grin2b* | N/A | N/A | 1.34* | 0.99 | 1.06 |
